# UAV Path Optimization for Target Passive Localization Considering the Position Uncertainty of the Target

**DOI:** 10.3390/s26175393

**Published:** 2026-08-26

**Authors:** Jiahao Lin, Liuhongye Song, Yuxiang Lu, Xueting Li, Wei Li, Genjiu Xu

**Affiliations:** 1 School of Aeronautics and Astronautics, Sichuan University, Chengdu 610065, China; 2023141510043@stu.scu.edu.cn (J.L.); 2023141510041@stu.scu.edu.cn (L.S.); 2023141510032@stu.scu.edu.cn (Y.L.); li.wei@scu.edu.cn (W.L.); 2 School of Mathematics and Statistics, Northwestern Polytechnical University, Xi’an 710072, China; xugenjiu@nwpu.edu.cn

**Keywords:** unmanned aerial vehicles (UAVs), target passive localization, target-position uncertainty, global Cramer–Rao lower bound (CRLB), analytical approximation, PSO

## Abstract

For the application of unmanned aerial vehicle (UAV)-based passive target localization, the positions of the UAVs play an important role because different UAV configurations provide distinct TDOA measurement geometries. In addition, the uncertainty of the target position affects the localization performance of different UAV configurations. Focusing on the problem of target localization by UAVs, this paper studies a UAV path optimization method for passive target localization considering target-position uncertainty. First, a passive localization signal model is established, and the TDOA method based on the Chan algorithm is deployed for target passive localization. Second, the Cramer–Rao lower bound (CRLB) for the Chan–TDOA localization method is derived as the criterion of the UAVs’ path optimization. To consider target-position uncertainty, the global CRLB is calculated within the uncertainty region of the target position instead of only applying the traditional single-point CRLB. Third, to improve computational efficiency, an analytical approximation of the global CRLB is derived from a second-order Taylor expansion instead of repeatedly calculating the multiple integral terms. By combining this objective with the PSO algorithm, the UAVs’ configuration is searched and applied at each time step. Finally, numerical simulations are performed to verify the validity and effectiveness of the proposed analytical global CRLB path-optimization method.

## 1. Introduction

As global technological advancements accelerate, modern warfare has evolved from traditional domains, such as land, sea, and air, to encompass diverse fields including the electromagnetic spectrum, cyberspace, outer space, as well as psychological and informational domains. According to the U.S. Joint Operations Manual JP 3-13.1 on Electronic Warfare, electronic warfare is defined as “the use of electromagnetic energy and directed energy to control the electromagnetic spectrum or to attack enemy military operations,” which includes electronic countermeasures, electronic protection, and electronic support [1]. In contemporary electronic warfare scenarios, target-position information constitutes critical intelligence for electronic reconnaissance systems, making accurate target localization essential for controlling the electromagnetic spectrum and executing precise strikes.

Generally, target localization methods can be classified into active and passive methods based on whether electromagnetic signals are actively transmitted. Active localization employs devices such as radars, lasers, and sonars to emit signals and capture their reflections from the targets, thereby extracting parameters to achieve more precise and more stable localization. However, the high transmitting power and the fixed frequency characteristics make it vulnerable to enemy detection and interference, particularly in the presence of advanced countermeasures such as low-altitude penetration, integrated electronic jamming, electromagnetic stealth, and anti-radiation missiles. In these scenarios, the active localization not only exhibits diminished localization accuracy in complex electromagnetic environments but also encounters significant threats to its operational security [2,3]. Consequently, it is imperative to develop target localization technologies which are better adapted to the demands of modern warfare.

In contrast, the target passive localization method obtains a target’s position by receiving signals emitted by the target or by capturing naturally occurring phenomena (e.g., electromagnetic, acoustic, or optical waves) induced by it, without employing any active transmission. Thus, the target passive localization method offers advantages in concealment and anti-jamming, making it particularly suitable for military and covert reconnaissance applications [4,5].

### 1.1. Overview

In this paper, we investigate the problem of target passive localization technology. Classified by the number of stations, two main technical approaches are prevalent: the single-station passive localization method [6] and the multi-station passive localization method [7].

Single-station passive localization systems have been extensively researched and applied in fields such as electronic reconnaissance and battlefield surveillance, owing to their high concealment and flexible deployment. The fundamental principle of such systems involves receiving signals from target positions via a single observation platform (e.g., UAVs, ground monitoring stations), extracting multi-dimensional feature information from the signals, combining this information with the platform’s own motion state to construct a localization model, and subsequently acquiring the target’s position [8]. More specifically, the system can extract critical information including the direction of arrival (DOA) and its rate of change [9], carrier frequency Doppler shift, dynamic characteristics of signal modulation, as well as the rate of change in signal intensity and time difference of arrival (TDOA). For example, the relative motion between the target and the observation platform not only causes dynamic changes in Doppler frequency shift but also continuously shifts the arrival direction angle over the localization process. By continuously collecting and analyzing these time-varying parameters, a system of kinematic equations encompassing variables such as azimuth, velocity, and distance can be established.

In contrast, multi-station passive localization systems employ multiple observation platforms collaboratively, integrating various types of target signal information (e.g., time-of-arrival (TOA) [10], time-difference-of-arrival (TDOA) [11], and angle-of-arrival (AOA) [12]) to localize targets. Research related to this approach focuses on synchronizing measurements across multiple stations, calibrating errors, and optimizing localization algorithms to further enhance the localization accuracy and the system stability.

Compared to single-station localization, multi-station passive localization offers significant benefits. A multi-station system can observe a target from multiple directions, thereby collecting richer data and effectively reducing the localization errors that usually arise in single-station scenarios due to limited observation angles or signal occlusion. This approach substantially enhances both the accuracy and reliability of passive localization, particularly in complex environments. Consequently, our paper focuses on exploring the multi-station passive localization methods. In such systems, the positioning of the UAVs is critical to achieving accurate target localization results. Accordingly, numerous scholars have devoted considerable effort to optimizing the UAVs’ configurations and optimizing the UAVs’ moving paths to further improve localization accuracy.

Significant research progress has been made in the field of UAV cooperative path optimization. For example, the study in [13] proposed an enhanced cheetah optimization algorithm to address spatial coordination constraints, temporal coordination constraints, and performance constraints in multi-UAV cooperative trajectory planning. The research in [14] integrates PSO with fast matching squared (FM^2^) technology to develop a PSO-FM^2^ hybrid algorithm, which dynamically adapts to avoid collisions and minimize the cost function, thereby optimizing multi-UAV path optimization in wireless networks. To achieve faster convergence and more efficient exploration of the global solution space, a multi-objective ant optimizer establishes a multi-objective optimization model and integrates adaptive random sequence mechanisms with directional evolution strategies [15]. Additionally, reinforcement learning algorithms that optimize the nearest strategy of multiple agents have been applied to dynamic target tracking and obstacle avoidance, improving the collaborative tracking accuracy of the UAVs and reducing latency [16]. A multi-objective multi-universe optimizer integrating multi-objective optimization and parallel computing can enhance the real-time response capability and robustness of path optimization in environments with dynamic obstacles [17].

Recent UAV semi-physical simulation platforms and scenario-driven path-planning evaluations further show the importance of testing UAV planning algorithms under realistic platform, navigation, energy, and environmental constraints [18,19].

Several recent studies are more directly related to localization-oriented UAV trajectory design. Li et al. optimize a UAV-swarm track using a hybrid TDOA/FDOA position–velocity model and an A-optimality criterion, while Dai et al. consider asynchronous three-dimensional passive multi-target tracking with collaborative multi-UAV trajectory optimization [20,21]. Chen et al. formulate variable-speed CRLB-based path optimization, whereas Xing et al. combine a planar Chan–TDOA model, a CRLB objective, and PSO-based path planning with node selection and no-fly-zone constraints [22,23]. The present study retains the core TDOA/CRLB trajectory-planning structure but considers TDOA-only target-position estimation, a fixed-speed heading decision, minimum inter-UAV spacing in place of a no-fly-zone constraint, and a probability-weighted global CRLB that explicitly accounts for target-position uncertainty.

However, current research on UAV path optimization generally assumes the target positions to be deterministic, an overly idealistic scenario that diverges significantly from real-world conditions. In practical environments, the target positions are usually affected by various factors, including measurement noise and signal interference, which introduce uncertainty. Neglecting the uncertainty of the target position may result in inaccurate optimized UAV trajectory, which is not suitable for the real situation, thereby compromising target localization accuracy. Consequently, recent studies have increasingly focused on addressing the challenges posed by target position uncertainty.

For instance, some studies have proposed the CWLS approximate iterative algorithm [24], which transforms the nonlinear relationship between the target position and auxiliary variables into a constrained quadratic programming problem. This approach employs linear approximate iteration to effectively address TDOA observation noise and sensor location errors in multi-target localization. The research in [25] introduces two semi-definite programming (SDP) methods: SDP-1, which optimizes both source and sensor locations concurrently, and SDP-2, which reallocates the uncertainty from the sensor location to the source location to reduce the computational complexity of the RSS method. Another study [26] examines the uncertainty associated with the localization of airborne external target positions and introduces the DP-SA algorithm, which effectively corrects covariance in real time to minimize localization errors in environments with significant clutter. Additionally, research in [27] indicates that the negative power log-likelihood loss function quantifies the direction of localization uncertainty; when integrated with a gray wolf optimizer, this approach improves the robustness of TDOA localization.

More specifically, Qu et al. [24] focus on multi-source passive localization in the presence of TDOA observation noise and sensor location errors, while Wang and Li [25] address RSS-based source localization with sensor-position uncertainty through SDP formulations. These works improve localization estimation under uncertain measurements or sensor positions. By contrast, the present study uses target-position uncertainty distribution to construct a global CRLB objective for UAV TDOA trajectory optimization and derives an analytical surrogate that can be evaluated efficiently during path planning.

The existing literature indicates that most studies address the target-position uncertainty problem separately, without combining it with UAV path optimization. Since the target-position uncertainty affects the localization performance of different UAV configurations at a large scale, ignoring this uncertainty in the planning objective may constrain the achievable localization accuracy.

To address this gap, the present study makes two specific contributions. First, target-position uncertainty is incorporated into TDOA-based UAV path planning through a probability-weighted global CRLB objective, rather than evaluating the local CRLB only at a single estimated target position. This formulation uses a normalized truncated Gaussian model over a finite computational support and is intended to improve planning performance and robustness when the target position supplied to the planner is uncertain.

Second, to avoid repeatedly evaluating the spatial integral during every candidate assessment, a second-order analytical surrogate is constructed using a Taylor expansion of the inverse-FIM trace, the inverse-matrix differential identity, and a covariance–Hessian contraction. These are standard mathematical tools; the methodological contribution is their specialization to the probability-weighted TDOA CRLB objective and their integration into the constrained PSO planning loop.

### 1.2. Original Contribution

The Chan–TDOA estimator and PSO solver are standard components used as the localization and search foundations of this study. Their contributions concern the uncertainty-aware planning objective and its efficient evaluation:

(1) Target-position uncertainty is incorporated into the UAV path-planning criterion. Specifically, the traditional single-point CRLB objective is replaced by a probability-weighted global CRLB over a normalized truncated Gaussian target-position distribution. This objective evaluates candidate UAV geometries over the target positions that may occur, thereby improving localization performance and reducing performance degradation as the target-position error increases.
(2) A second-order analytical approximation is derived to reduce the repeated integration cost of the global CRLB inside the trajectory optimizer. The inverse-FIM trace is expanded at the estimated target position; the first-order term vanishes under the symmetric zero-mean model; and the remaining second-order expectation is expressed as a covariance–Hessian contraction. Embedding this scalar surrogate into each constrained PSO candidate evaluation provides a computationally efficient approximation while preserving the principal performance benefit of the MC global-CRLB reference.

### 1.3. Article Organization

The main content of this paper is presented as follows. Section 2 establishes the target passive localization signal model of the Chan–TDOA algorithm. Section 3 formulates the problem of UAV path optimization considering the target position uncertainty. To accelerate the UAV path optimization algorithm, the analytical expression of the global CRLB is derived instead of directly calculating the multiple integral terms, and then the PSO algorithm is applied to search for the optimal UAV trajectory in Section 4. Numerical simulations are provided in Section 5, and the paper is concluded in Section 6.

## 2. Target Passive Localization Signal Model Based on the Chan–TDOA Method

**Figure 1:**
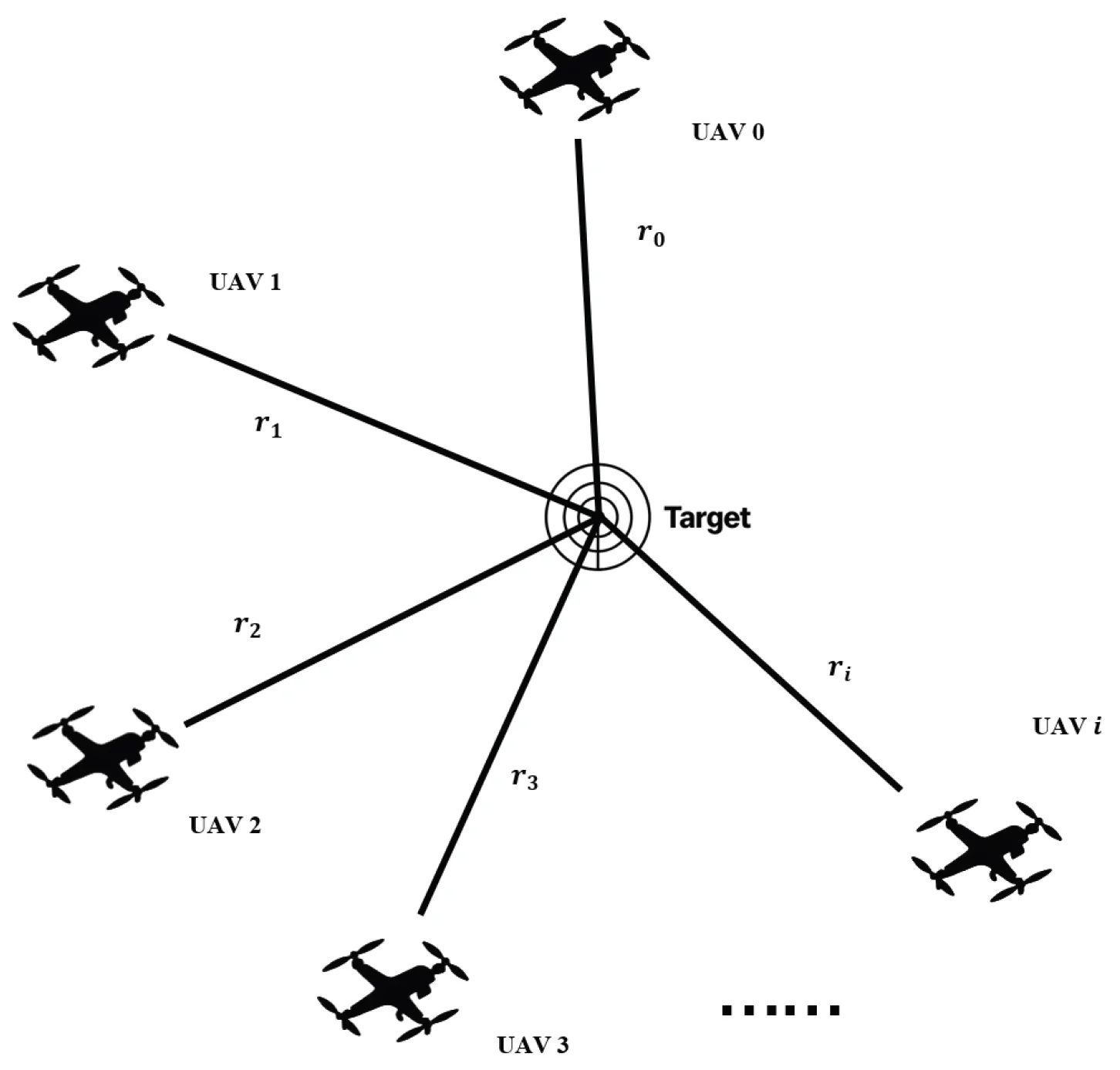
The geometric schematic of the Chan–TDOA localization model. UAV0 serves as the reference (main) UAV, while UAV1, UAV2, and UAV3 illustrate representative non-reference nodes; UAV *i* denotes any other node with i=1,…,I−1. The target-to-UAV distances are denoted by $r_{0}$, $r_{1}$, $r_{2}$, $r_{3}$, and, in the general notation, $r_{i}$.

In this part, we establish a basic Chan–TDOA localization signal model. Consider a two-dimensional target passive localization scenario where multiple UAVs and a target exist as illustrated in Figure 1. The horizontal-plane formulation follows the original problem setting and is applicable when the UAV altitude and the target altitude are known, fixed, or controlled separately. The corresponding 3D extension can be obtained by augmenting the position vector, the TDOA Jacobian, and the FIM with the altitude coordinate.

Assume that the target is located at z=[x,y]. The coordinates of the receiving stations for *I* UAVs are given by $w_{i}=\left[x_{i},y_{i}\right]$, where i=0,1,…,I−1. Here, we select UAV0 $\left(x_{0},y_{0}\right)$ as the main UAV. The distance from the target position to the *i*th UAV is

(1) $$r_{i}=∥w_{i}-z∥=\sqrt{{\left(x_{i}-x\right)}^{2}+{\left(y_{i}-y\right)}^{2}},\;i=0,1,\ldots ,I-1$$

The time taken for the target signal to reach the *i*th UAV is denoted as $\tau_{i}$. The time difference between the signal’s arrival at the *i*th UAV and the main UAV (UAV0) is denoted as $\tau_{i,0}$. The line-of-sight propagation model and additive Gaussian TDOA-error abstraction used below are standard idealized models in recent TDOA localization and UAV trajectory-optimization studies [20,22,23,28,29]. Neglecting non-line-of-sight bias and multipath effects, we obtain the following equation

(2) $$\tau_{i,0}=\tau_{i}-\tau_{0}=\frac{r_{i}-r_{0}}{c}\left(i=1,2,\ldots ,I-1\right)$$

where c represents the propagation speed of an electromagnetic wave. By multiplying both sides of Equation (2) by *c*, the distance difference between the target from UAV0 and *i*th UAV can be described as [30].

(3) $$\Delta r_{i,0}=c\cdot \tau_{i,0}=r_{i}-r_{0}$$

Additionally, to eliminate the ambiguity of the estimated target position, a minimum of three UAVs are needed to acquire sufficient TDOA measurements for two-dimensional target localization. The nonlinear equations for the TDOA target localization method can be derived from the measurements as follows

(4) $$\left\{\begin{array}{c} r_{0}=\sqrt{{\left(x-x_{0}\right)}^{2}+{\left(y-y_{0}\right)}^{2}}\\ r_{i}=\sqrt{{\left(x-x_{i}\right)}^{2}+{\left(y-y_{i}\right)}^{2}}\\ \Delta r_{i,0}=c\cdot \tau_{i,0} \end{array}\right.$$

In practical applications, the measurement $\tau_{i,0}^{\prime }$ usually exhibits measurement errors which can be described as

(5) $$\tau_{i,0}^{\prime }=\tau_{i,0}+\varepsilon_{i,0}$$

where $\tau_{i,0}^{\prime }$ denotes the actual measured time difference, $\tau_{i,0}$ represents the true time difference, and $\varepsilon_{i,0}$ is the measurement error, which generally follows a Gaussian noise distribution

(6) $$\varepsilon_{i,0}∼N\left(0,\sigma_{i,0}^{2}\right)$$

where $\sigma_{i,0}^{2}=\sigma_{i}^{2}+\sigma_{0}^{2}$ represents the measurement error variance of the target, while $\sigma_{i}^{2}$ and $\sigma_{0}^{2}$ denote the measurement variances of UAV *i* and UAV 0, respectively. Substituting Equation (6) into Equation (1), we can obtain

(7) $$r_{i}-r_{0}=c\cdot \tau_{i,0}^{\prime }-c\cdot \varepsilon_{i,0}$$

and squaring both sides of the equation yields the following expression

(8) $$r_{i}^{2}={\left(r_{0}+c\cdot \tau_{i,0}^{\prime }-c\cdot \varepsilon_{i,0}\right)}^{2}$$

Under the assumption that the measurement error, denoted as $c\cdot \varepsilon_{i,0}$, is relatively small, the above formula can be written while neglecting the quadratic term

(9) $$r_{i}^{2}\approx {\left(r_{0}+c\cdot \tau_{i,0}^{\prime }\right)}^{2}-2\left(r_{0}+c\cdot \tau_{i,0}^{\prime }\right)\left(c\cdot \varepsilon_{i,0}\right)$$

Substituting $r_{i}^{2}={\left(x-x_{i}\right)}^{2}+{\left(y-y_{i}\right)}^{2}$ into Equation (9), and $r_{0}^{2}={\left(x-x_{0}\right)}^{2}+{\left(y-y_{0}\right)}^{2}$, the pseudo-linear equations concerning the unknown target position $\left(x,y\right)$ and the auxiliary variable $r_{0}$ can be obtained

(10) $$2\left(x_{0}-x_{i}\right)x+2\left(y_{0}-y_{i}\right)y-2\left(c\cdot \tau_{i,0}^{\prime }\right)r_{0}=\left(x_{0}^{2}-x_{i}^{2}\right)+\left(y_{0}^{2}-y_{i}^{2}\right)+{\left(c\cdot \tau_{i,0}^{\prime }\right)}^{2}-\psi_{i}$$

where

(11) $$\psi_{i}\approx 2\left(r_{0}+c\cdot \tau_{i,0}^{\prime }\right)\left(c\cdot \varepsilon_{i,0}\right)\approx 2r_{i}\left(c\cdot \varepsilon_{i,0}\right)$$

is the error term introduced by linearization. Combining I−1 equations to construct the matrix form of the pseudo-linear equations, we can obtain

(12) Gs=h−ψ

where

(13) $$G=\left[\begin{array}{ccc} x_{0}-x_{1} & y_{0}-y_{1} & -c\cdot \tau_{1,0}^{\prime }\\ \vdots & \vdots & \vdots \\ x_{0}-x_{I-1} & y_{0}-y_{I-1} & -c\cdot \tau_{I-1,0}^{\prime } \end{array}\right]$$

(14) $$h=\frac{1}{2}\left[\begin{array}{c} \left(x_{0}^{2}-x_{1}^{2}\right)+\left(y_{0}^{2}-y_{1}^{2}\right)+{\left(c\cdot \tau_{1,0}^{\prime }\right)}^{2}\\ \vdots \\ \left(x_{0}^{2}-x_{I-1}^{2}\right)+\left(y_{0}^{2}-y_{I-1}^{2}\right)+{\left(c\cdot \tau_{I-1,0}^{\prime }\right)}^{2} \end{array}\right]$$

and the ψ is

(15) $$\psi ={\left[\psi_{1},\ldots ,\psi_{I-1}\right]}^{T}$$

The covariance matrix can be approximated as

(16) $$\mathrm{Cov}\left(\psi \right)\approx 4c^{2}BQB$$

To enhance the efficiency of solving Equation (12), the Chan algorithm is introduced in this paper. Through a clever linearization transformation and a two-step weighted least squares (WLS) estimation, the algorithm provides an efficient closed-form solution for the position of the target.

The first step of the WLS estimation is to solve the above pseudo-linear equations

(17) $$J_{1}={\left(h-\mathrm{Gs}\right)}^{T}W_{1}\left(h-\mathrm{Gs}\right)$$

where $W_{1}$ is the inverse of the covariance matrix of the error vector ψ, i.e.,

(18) $$W_{1}=\mathrm{Cov}{\left(\psi \right)}^{-1}$$

where

(19) $$B=\mathrm{diag}\{r_{1},\ldots ,r_{I-1}\}$$

In practical Chan implementation, the true target-to-UAV ranges in B are not assumed to be known. A preliminary position estimate is first obtained from the pseudo-linear equations using an identity or approximate weighting matrix, and the corresponding estimated ranges are then used to construct B and update the WLS weighting matrix [31]. The simulations in this paper use the Chan localization algorithm as the localization module.

ε is the TDOA measurement error vector

(20) $$\varepsilon ={\left[\varepsilon_{1,0},\ldots ,\varepsilon_{I-1,0}\right]}^{T}$$

and Q is the covariance matrix of the error vector ε.

(21) $$Q=E\left[\epsilon \epsilon^{T}\right]=\left[\begin{array}{cccc} \sigma_{1,0}^{2} & \sigma_{0}^{2} & \ldots & \sigma_{0}^{2}\\ \sigma_{0}^{2} & \sigma_{2,0}^{2} & \ldots & \sigma_{0}^{2}\\ \vdots & \vdots & \ddots & \vdots \\ \sigma_{0}^{2} & \sigma_{0}^{2} & \ldots & \sigma_{I-1,0}^{2} \end{array}\right]$$

Using the WLS method, a preliminary estimate of s, where the bracketed superscript (1) is defined to denote the first-step of the estimation, can be obtained as

(22) $${\hat{s}}^{\left(1\right)}={\left(G^{T}W_{1}G\right)}^{-1}G^{T}W_{1}h$$

To enhance the accuracy of the localization result, a second-step WLS estimation is performed. Let the estimation error from the first step be $\delta s^{\left(1\right)}={\hat{s}}^{\left(1\right)}-s$, with its covariance matrix evaluated as

(23) $$C_{s}=\mathrm{Cov}\left({\hat{s}}^{\left(1\right)}\right)={\left(G^{T}W_{1}G\right)}^{-1}G^{T}W_{1}\mathrm{Cov}\left(\psi \right)W_{1}G{\left(G^{T}W_{1}G\right)}^{-1}$$

The unknown parameter vector is redefined

(24) $$s^{\prime }={\left[{\left(x-x_{0}\right)}^{2},{\left(y-y_{0}\right)}^{2}\right]}^{T}$$

the linear relationship of the second stage is constructed as

(25) $$h^{\prime }=G^{\prime }s^{\prime }+\psi^{\prime }$$

where

(26) $$h^{\prime }={\left[{\left({\hat{x}}^{\left(1\right)}-x_{0}\right)}^{2},{\left({\hat{y}}^{\left(1\right)}-y_{0}\right)}^{2},{\left({\hat{r}}_{0}^{\left(1\right)}\right)}^{2}\right]}^{T}$$

(27) $$G^{\prime }=\left[\begin{array}{cc} 1 & 0\\ 0 & 1\\ 1 & 1 \end{array}\right]$$

and ${\hat{x}}^{\left(1\right)}$, ${\hat{y}}^{\left(1\right)}$, and ${\hat{r}}_{0}^{\left(1\right)}$ are extracted from ${\hat{s}}^{\left(1\right)}$.

The covariance matrix of the new error vector $\psi^{\prime }$ is

(28) $$C_{\psi^{\prime }}\approx B^{\prime }C_{s}{\left(B^{\prime }\right)}^{T}$$

where $B^{\prime }$ is given by

(29) $$B^{\prime }=2\cdot \mathrm{diag}\left\{{\hat{x}}^{\left(1\right)}-x_{0},{\hat{y}}^{\left(1\right)}-y_{0},{\hat{r}}_{0}^{\left(1\right)}\right\}$$

The WLS localization estimate can be obtained as

(30) $${\hat{s}}^{\prime }={\left({\left(G^{\prime }\right)}^{T}C_{\psi^{\prime }}^{-1}G^{\prime }\right)}^{-1}{\left(G^{\prime }\right)}^{T}C_{\psi^{\prime }}^{-1}h^{\prime }$$

Finally, the estimated position of the target is computed, yielding $\hat{x}=\pm \sqrt{{\left[{\hat{s}}^{\prime }\right]}_{1}}+x_{0}$ and $\hat{y}=\pm \sqrt{{\left[{\hat{s}}^{\prime }\right]}_{2}}+y_{0}$.

## 3. UAV Path Optimization Algorithm Based on the Global CRLB

### 3.1. Problem Description

At time *t*, *I* UAVs perform the target passive localization task, and the path optimization algorithm is updated with the time interval of Δt. For simplicity, each UAV is assumed to move at a constant velocity *v* during the path optimization process. The key variable to optimize is the deflection angle $\phi_{i}^{t}$ (i=0,1,2,…,I−1), which is defined as the angle between the heading direction of the UAVs and the positive x-axis. By optimizing $\phi_{i}^{t}$, the waypoint position of each UAV at the next moment (t+1) can be uniquely determined; thus, optimizing $\phi_{i}^{t}$ is equivalent to directly optimizing the UAV’s spatial position at a later time. Let the UAV’s position set at time *t* be denoted by $U^{t}={\left\{w_{i}^{t}\right\}}_{i=0}^{I-1}$. $w_{i}^{t}=\left[x_{i}^{t},y_{i}^{t}\right]$ denotes the two-dimensional coordinates of the *i*th UAV at time *t*.

### 3.2. Target Position Uncertainty Modeling

The position estimation of the target is related to the target-position uncertainty. It is assumed that the true position of the target is denoted as $z^{*}$, following a two-dimensional Gaussian distribution centered at $\hat{z}$.

(1) Probabilistic Distribution Assumption

Assuming that the difference between the estimated position $\hat{z}$ and the true position $z^{*}$ of the target at time *t* follows a normal distribution $N(0_{2\times 1},M_{2\times 2})$, we obtain that

(31) $$f\left({\hat{z}}^{t};z^{*}\right)=\frac{1}{2\pi \sqrt{\left|M\right|}}\exp\left(-\frac{1}{2}{\left({\hat{z}}^{t}-z^{*}\right)}^{T}M^{-1}\left({\hat{z}}^{t}-z^{*}\right)\right)$$

M denotes the covariance matrix, given by

(32) $$M=\left[\begin{array}{cc} \sigma_{x}^{2} & \sigma_{xy}\\ \sigma_{xy} & \sigma_{y}^{2} \end{array}\right]$$

where $\sigma_{x}^{2}$ and $\sigma_{y}^{2}$ represent the error variances in the x-axis and the y-axis, respectively.

(2) Uncertain Integration Region

To account for the position uncertainty of the target, a square uncertain region is defined with its center at $\left(\hat{x},\hat{y}\right)$ and with boundaries given by

(33) $$x\in \left[\hat{x}-\frac{A}{2},\;\hat{x}+\frac{A}{2}\right],\;y\in \left[\hat{y}-\frac{A}{2},\;\hat{y}+\frac{A}{2}\right]$$

Here, the square region is used as a finite computational support, not as a uniform target distribution. Within this support, the Gaussian density in Equation (31) is normalized as a truncated Gaussian,

(34) $$\tilde{f}\left({\hat{z}}^{t};z^{*},\Phi \right)=\frac{f({\hat{z}}^{t};z^{*})}{{\;\int \int \;}_{\Phi }f\left({\hat{z}}^{t};\xi \right)d\xi },\;z^{*}\in \Phi .$$

Thus, the square defines the finite integration domain, whereas the probability weights remain Gaussian after normalization. This resolves the apparent inconsistency between the square uncertainty region and the Gaussian target-position uncertainty model.

This uncertainty model is related to, but not identical to, the probabilistic models used in earlier UAV planning studies. Dogancay and Hmam use Gaussian priors and a recursive Bayesian FIM for single-UAV angle-only self-localization and target tracking, whereas Li et al. optimize multistatic antenna deployment when the moving radar-platform positions follow a uniform uncertainty model [32,33]. In contrast, the random quantity in the present study is the target position supplied to a multi-UAV TDOA planner, and the planning objective is the probability-weighted global CRLB over a normalized truncated Gaussian support.

Here, the side length *A* of the region is dynamically adjusted based on the average distance from three UAVs to the target’s initial position $\left({\hat{x}}_{\mathrm{init}},{\hat{y}}_{\mathrm{init}}\right)$, computed as

(35) $$r_{avg}=\frac{\left(\sqrt{{\left(x_{0}-{\hat{x}}_{\mathrm{init}}\right)}^{2}+{\left(y_{0}-{\hat{y}}_{\mathrm{init}}\right)}^{2}}+\cdots +\sqrt{{\left(x_{I-1}-{\hat{x}}_{\mathrm{init}}\right)}^{2}+{\left(y_{I-1}-{\hat{y}}_{\mathrm{init}}\right)}^{2}}\right)}{I}$$

Then, the size of the uncertain area can be determined as follows

(36) $$A=\min\left(A_{max},A_{min}+\kappa \cdot r_{avg}\right)$$

In order to avoid the system error in calculating the global CRLB, the minimum side length is determined as $A_{\min}=6\sigma$ to guarantee that the uncertain integration area covers the effective probability mass of the Gaussian distribution, and the maximum side length is determined as $A_{\max}=10\sigma$ to guarantee that when the UAVs are far from the target, the uncertain integration area adequately encompasses the high-probability region of the target’s real position.

### 3.3. CRLB Derivation for the Chan–TDOA Localization Algorithm

The CRLB is a critical metric evaluating the performance of parameter estimation. It defines the minimum variance that an unbiased estimator can achieve, and can be derived from the inverse of the Fisher information matrix (FIM).

For the target passive localization application based on the Chan–TDOA algorithm formulated above, the measurements from the target can be represented as

(37) $$m=c{\left[\tau_{1,0},\tau_{2,0},\ldots ,\tau_{I-1,0}\right]}^{T}$$

and the measurement value can be expressed as

(38) $$m^{\prime }=m+\varepsilon$$

where the measurement error ε follows a multivariate normal distribution N(0,Q). Consider the measurement ε, the measurements in the real situation can be described as

(39) $$Q=E[\varepsilon \varepsilon^{T}]$$

Thus, the probability density function of the Chan–TDOA measurements can be expressed as

(40) $$p\left(m^{\prime }|m\right)=\frac{1}{\sqrt{2\pi |Q|}}\exp\left(-\frac{1}{2}{\left(m^{\prime }-m\right)}^{T}Q^{-1}\left(m^{\prime }-m\right)\right).$$

The likelihood function can be obtained as follows

(41) $$\ln\left(p(m^{\prime }|m)\right)=-\frac{\ln|2\pi Q|}{2}-\frac{{\left(m^{\prime }-m\right)}^{T}Q^{-1}\left(m^{\prime }-m\right)}{2}$$

Taking the second partial derivatives of Equation (41), we have

(42) $$\begin{array}{cc} \frac{\partial^{2}\ln p\left(m^{\prime }|m\right)}{\partial x^{2}}= & -\frac{1}{2}[{\left(-\frac{\partial^{2}m}{\partial x^{2}}\right)}^{T}Q^{-1}\left(m^{\prime }-m\right)\\ & -{\left(m^{\prime }-m\right)}^{T}Q^{-1}\frac{\partial^{2}m}{\partial x^{2}}+2{\left(\frac{\partial m}{\partial x}\right)}^{T}Q^{-1}\frac{\partial m}{\partial x}] \end{array}$$

(43) $$\begin{array}{cc} \frac{\partial^{2}\ln p\left(m^{\prime }|m\right)}{\partial x\partial y}= & -\frac{1}{2}[{\left(-\frac{\partial^{2}m}{\partial x\partial y}\right)}^{T}Q^{-1}\left(m^{\prime }-m\right)\\ & -{\left(m^{\prime }-m\right)}^{T}Q^{-1}\frac{\partial^{2}m}{\partial x\partial y}+2{\left(\frac{\partial m}{\partial x}\right)}^{T}Q^{-1}\frac{\partial m}{\partial y}] \end{array}$$

(44) $$\begin{array}{cc} \frac{\partial^{2}\ln p\left(m^{\prime }|m\right)}{\partial y^{2}}= & -\frac{1}{2}[{\left(-\frac{\partial^{2}m}{\partial y^{2}}\right)}^{T}Q^{-1}\left(m^{\prime }-m\right)\\ & -{\left(m^{\prime }-m\right)}^{T}Q^{-1}\frac{\partial^{2}m}{\partial y^{2}}+2{\left(\frac{\partial m}{\partial y}\right)}^{T}Q^{-1}\frac{\partial m}{\partial y}] \end{array}$$

The expected value of Equations (42)–(44) can be calculated as follows

(45) $$E\left[\frac{\partial^{2}\ln p\left(m^{\prime }|m\right)}{\partial x^{2}}\right]=-\left[{\left(\frac{\partial m}{\partial x}\right)}^{T}Q^{-1}\frac{\partial m}{\partial x}\right]$$

(46) $$E\left[\frac{\partial^{2}\ln p\left(m^{\prime }|m\right)}{\partial x\partial y}\right]=-\left[{\left(\frac{\partial m}{\partial x}\right)}^{T}Q^{-1}\frac{\partial m}{\partial y}\right]$$

(47) $$E\left[\frac{\partial^{2}\ln p\left(m^{\prime }|m\right)}{\partial y^{2}}\right]=-\left[{\left(\frac{\partial m}{\partial y}\right)}^{T}Q^{-1}\frac{\partial m}{\partial y}\right]$$

The Fisher information matrix of the target position parameters, can be expressed as

(48) $$F\left(m\right)=-\left[\begin{array}{cc} E\left[\frac{\partial^{2}\ln p\left(m^{\prime }|m\right)}{\partial x^{2}}\right] & E\left[\frac{\partial^{2}\ln p\left(m^{\prime }|m\right)}{\partial x\partial y}\right]\\ E\left[\frac{\partial^{2}\ln p\left(m^{\prime }|m\right)}{\partial x\partial y}\right] & E\left[\frac{\partial^{2}\ln p\left(m^{\prime }|m\right)}{\partial y^{2}}\right] \end{array}\right]=\left[\begin{array}{cc} {\left(\frac{\partial m}{\partial x}\right)}^{T}Q^{-1}\frac{\partial m}{\partial x} & {\left(\frac{\partial m}{\partial x}\right)}^{T}Q^{-1}\frac{\partial m}{\partial y}\\ {\left(\frac{\partial m}{\partial x}\right)}^{T}Q^{-1}\frac{\partial m}{\partial y} & {\left(\frac{\partial m}{\partial y}\right)}^{T}Q^{-1}\frac{\partial m}{\partial y} \end{array}\right]$$

Therefore, by taking the inverse of the Fisher information matrix, the CRLB matrix can be expressed as

(49) $$\mathrm{CRLB}\left(m\right)=F^{-1}\left(m\right)$$

### 3.4. Global CRLB Derivation Considering Target Position Uncertainty

In this paper, we propose a UAV path optimization method based on the CRLB. To consider the uncertainty of the target position, we use the global CRLB within the uncertainty area of target position instead of the traditional single-point CRLB.

**Figure 2:**
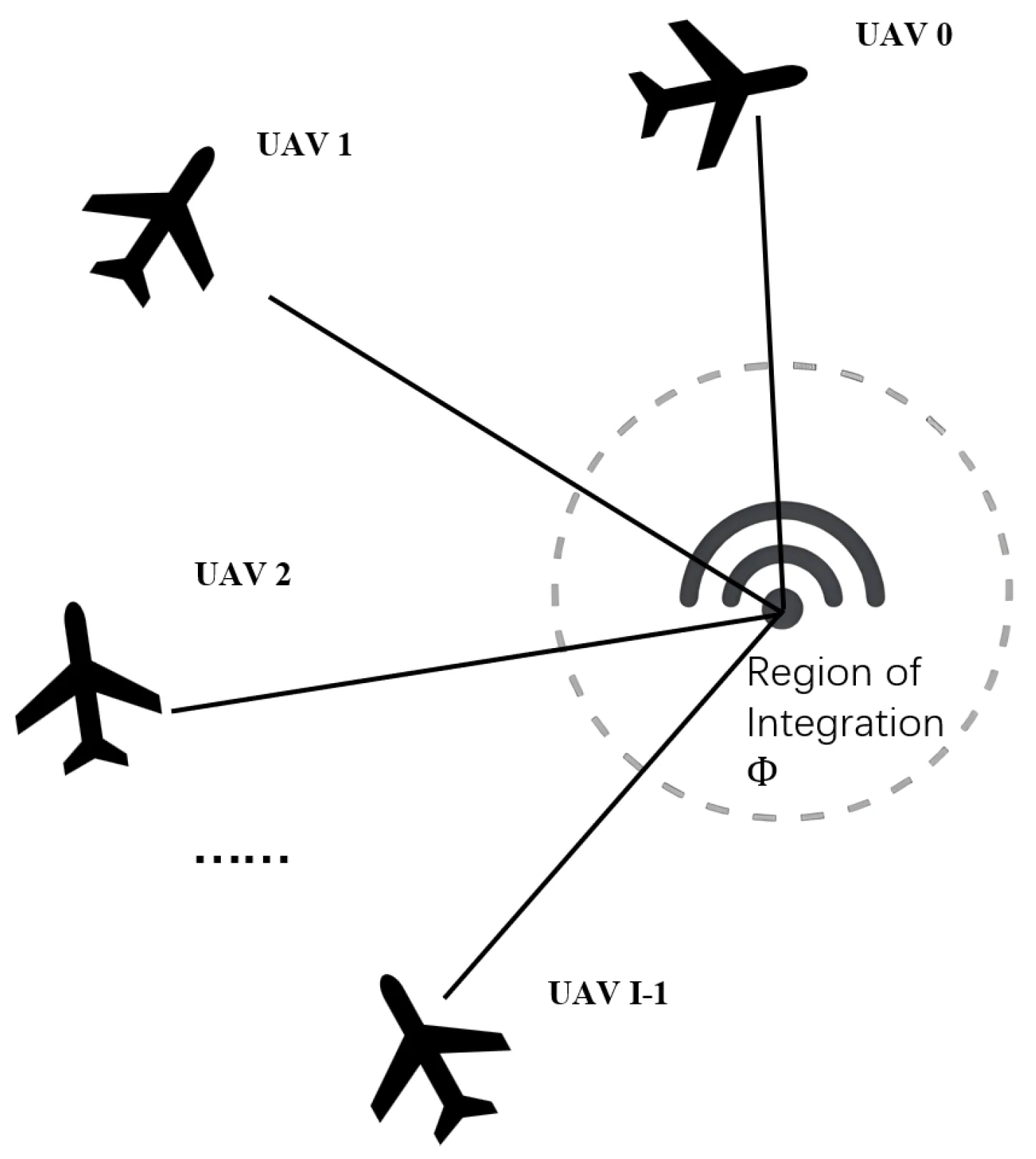
Schematic diagram of the UAVs’ passive localization based on the global CRLB algorithm proposed in this paper.

In the uncertain region Φ depicted in Figure 2, the likelihood that the true position of the target $z^{*}$ lies at any specific point can be quantitatively described by the probability density function in Equation (31). Based on Equation (49), we define the single-point CRLB matrix function $\mathrm{Cr}(U^{t};z^{*})$ as the inverse of the Fisher Information Matrix, which is inherently coupled with the UAVs’ spatial configuration $U^{t}$ and the true position of the target $z^{*}$

(50) $$\mathrm{Cr}\left(U^{t};z^{*}\right)={\left({\left[\begin{array}{c} {\left(\frac{z^{*}-w_{1}^{t}}{∥z^{*}-w_{1}^{t}∥}-\frac{z^{*}-w_{0}^{t}}{∥z^{*}-w_{0}^{t}∥}\right)}^{T}\\ \vdots \\ {\left(\frac{z^{*}-w_{I-1}^{t}}{∥z^{*}-w_{I-1}^{t}∥}-\frac{z^{*}-w_{0}^{t}}{∥z^{*}-w_{0}^{t}∥}\right)}^{T} \end{array}\right]}^{T}Q^{-1}\left[\begin{array}{c} {\left(\frac{z^{*}-w_{1}^{t}}{∥z^{*}-w_{1}^{t}∥}-\frac{z^{*}-w_{0}^{t}}{∥z^{*}-w_{0}^{t}∥}\right)}^{T}\\ \vdots \\ {\left(\frac{z^{*}-w_{I-1}^{t}}{∥z^{*}-w_{I-1}^{t}∥}-\frac{z^{*}-w_{0}^{t}}{∥z^{*}-w_{0}^{t}∥}\right)}^{T} \end{array}\right]\right)}^{-1}$$

By taking the integral of the single-point CRLB matrix formulated in Equation (50) within the uncertain region Φ, weighting by the normalized truncated spatial probability distribution $\tilde{f}\left({\hat{z}}^{t};z^{*},\Phi \right)$ in Equation (34), we obtain the global CRLB expression at time *t*

(51) $${\mathrm{Cr}}_{\Phi }^{t}=\underset{\Phi }{\;\int \int \;}\tilde{f}\left({\hat{z}}^{t};z^{*},\Phi \right)\cdot \mathrm{Cr}\left(U^{t};z^{*}\right)\;dz^{*}$$

Then, the objective function of the UAV path optimization problem can be obtained as follows

(52) $$J=\min_{w_{i}^{t+1}}\arg\mathrm{Tr}\left({\mathrm{Cr}}_{\Phi }^{t+1}\right)$$

### 3.5. Constraint Analysis

In this paper, we mainly consider four constraints for the UAV path optimization problem: the minimum turning radius, the minimum spacing distance, the minimum approach distance and the deflection angle constraint. These choices follow the general use of motion-feasibility and target-oriented trajectory constraints in localization-driven UAV planning [21,22,23,32]. More specifically, the present formulation retains turning, approach, and target-oriented heading constraints used in closely related CRLB-based planning frameworks, replaces the no-fly-zone constraint in Xing et al. [23] with a minimum inter-UAV spacing constraint, and uses a fixed-speed heading feasible set rather than the variable-speed feasible region in Chen et al. [22].

(1) **Turning radius limit:** The actual turning radius $R_{i}^{t}$ of a UAV must be greater than the minimum turning radius $R_{\min}$ which is determined by the UAV system parameters, i.e.,
  (53) $$R_{i}^{t}>R_{\min}$$
  Within the time interval Δt, the turning radius $R_{i}^{t}$ of each UAV can be calculated by the UAV’s movement. Assume that the UAVs move at a constant speed *v*, and the turning angle formed over this interval is denoted by $\theta_{i}^{t}$. Consider that the waypoint coordinates at successive time instances *t* and t+1 are denoted as $w_{i}^{t}$ and $w_{i}^{t+1}$, respectively, then the turning radius $R_{i}^{t}$ and the corresponding circular arc central angle $\theta_{i}^{t}$ can be derived as
  (54) $$\Delta d^{t}=\sqrt{{\left(x_{i}^{t+1}-x_{i}^{t}\right)}^{2}+{\left(y_{i}^{t+1}-y_{i}^{t}\right)}^{2}}=2R_{i}^{t}\sin\left(2\theta_{i}^{t}\right)$$
  where $\Delta d^{t}$ represents the Euclidean distance between two waypoints. Through Equation (54), we can solve the turning angle $\theta_{i}^{t}$ and the turning radius $R_{i}^{t}$.
(2) **Minimum Spacing Distance Constraint:** To avoid drone collisions between the UAVs, the distance $D_{i}^{t}$ between each UAV and any other must not be less than the minimum spacing distance $D_{\min}$; therefore, $D_{i}^{t}$ should satisfy
  (55) $$D_{i}^{t}>D_{\min}$$
(3) **Minimum Approach Distance Constraint:** To keep the UAVs outside the minimum approach range of the target, the distance $L_{i}^{t}$ between each UAV and the target must not be less than the minimum approach distance $L_{\min}$, i.e.,
  (56) $$L_{i}^{t}>L_{\min}$$
(4) **Deflection Angle Constraint:** To ensure that the UAVs move toward the target, we stipulate that at time *t*, the heading direction of UAV *i* must lie within the angular range defined by $\Delta \alpha_{i}^{t}$ relative to the line connecting it to the target. Accordingly, the deflection angle $\phi_{i}^{t}$ of UAV *i* at time *t* must satisfy
  (57) $$\phi_{i}^{t}\in \left(\alpha_{i}^{t}-\Delta \alpha_{\mathrm{target}},\alpha_{i}^{t}+\Delta \alpha_{\mathrm{target}}\right)$$
  where
  (58) $$\alpha_{i}^{t}=\arctan\frac{\hat{y}-y_{i}^{t}}{\hat{x}-x_{i}^{t}}$$
  In the equation, $\alpha_{i}^{t}$ denotes the target azimuth of the *i*th UAV at time *t*, $\hat{x}$ and $\hat{y}$ are the estimated coordinates of the target position $\hat{z}$, and $x_{i}^{t}$ and $y_{i}^{t}$ represent the current position coordinates of the *i*th UAV at time *t*.

### 3.6. UAV Path Optimization Mathematical Model

The mathematical model of the UAV path optimization problem can be described as follows, which contains the objective function and the constraints

(59) $$\begin{array}{cc} \; & \min_{w_{i}^{t+1}}\arg\mathrm{Tr}\left({\mathrm{Cr}}_{\Phi }^{t+1}\right)\\ \; & \mathrm{subject}\mathrm{to}\left\{\begin{array}{c} R_{i}^{t}>R_{\min}\\ D_{i}^{t}>D_{\min}\\ L_{i}^{t}>L_{\min}\\ \phi_{i}^{t}\in \left(\alpha_{i}^{t}-\Delta \alpha_{\mathrm{target}},\;\alpha_{i}^{t}+\Delta \alpha_{\mathrm{target}}\right) \end{array}\right.,\;i=0,1,2,\ldots ,I-1 \end{array}$$

## 4. Global CRLB Calculation and PSO Algorithm

### 4.1. Monte Carlo Calculation Solution

The calculation of the global CRLB is a critical theoretical foundation for the path optimization problem when the target position is uncertain. The mathematical expression of the global CRLB can be referred to in Equation (51), which is the weighted average of the single-point CRLBs in the uncertain region Φ where the target may exist. First, within area Φ, $K_{s}$ sampling points ${\left\{p_{\gamma }\right\}}_{\gamma =1}^{K_{s}}$ are generated to approximate the global CRLB integral. Next, for each sampling point, we calculate the single-point CRLB referred to in Equation (49) and its normalized truncated probability weight referred to in Equation (34). Then, the global CRLB approximation is obtained by summing all the sampling points’ weighted results shown as follows

(60) $${\mathrm{Cr}}_{\Phi }^{t}\approx \sum_{\gamma =1}^{K_{s}}\omega_{\gamma }\cdot \mathrm{Cr}\left(U^{t};p_{\gamma }\right),\;\omega_{\gamma }=\frac{\tilde{f}\left({\hat{z}}^{t};p_{\gamma },\Phi \right)}{\sum_{l=1}^{K_{s}}\tilde{f}\left({\hat{z}}^{t};p_{l},\Phi \right)}$$

When the sampling points are uniformly distributed and *c* is sufficiently large, according to the law of large numbers, the approximation will converge to the true global CRLB. Employing this Monte Carlo algorithm can effectively balance the complexity of multiple integrals with the rapid response requirements of the localization process, while maintaining high accuracy.

### 4.2. Analytical Calculation Solution

Referring to Equation (51), the global CRLB is based on a two-dimensional integration which contains mutual dependency and coupling effects. Therefore, the computation complexity is very high. Although the Monte Carlo algorithm can potentially calculate the true value of the global CRLB by gradually increasing the number of sample points, the required high sample size raises the computational load substantially.

The following derivation should be interpreted as an analytical surrogate for the probability-weighted global CRLB used in the UAV path optimizer. Here, “global CRLB” denotes the spatially probability-weighted average of conditional local CRLB matrices over the adopted target-position uncertainty region. The use of a second-order Taylor expansion and matrix differential identities follows standard approximation theory and standard matrix calculus. The contribution here is the specialization of this expansion to the trace of the inverse TDOA FIM under target-position uncertainty and its use as an efficient replacement for repeated Monte Carlo integration in the path-planning loop.

To improve the computation efficiency, in this paper, we propose an analytical calculation method to solve the complex integration terms by using the Taylor series expansion. To simplify the derivation process, we omit the time index *t* in this part to obtain the analytical expression of the global CRLB. The Taylor expansion for the trace of the single-point CRLB at the estimated target position $\hat{z}$ can be expressed as

(61) $$\mathrm{Tr}\left(F^{-1}\left(z\right)\right)\approx \mathrm{Tr}\left(F^{-1}\left(\hat{z}\right)\right)+\sum_{|\beta |=1}^{\infty }{\left.\frac{1}{\beta !}\frac{\partial^{\left|\beta \right|}\mathrm{Tr}\left(F^{-1}\left(z\right)\right)}{\partial z^{\beta }}\right|}_{\hat{z}}{\left(z-\hat{z}\right)}^{\beta }$$

where z is the target true location, and $\hat{z}$ is the estimated target position which is set as the Taylor expansion center. A multi-index notation β is applied to properly characterize the derivative behavior. For the problem of calculating the global CRLB, the second-order expansion can effectively meet the dual requirements of accuracy and computational efficiency for online UAV path optimization, and then we can rewrite Equation (61) as

(62) $$\begin{array}{cc} \mathrm{Tr}(F^{-1}\left(z\right)) & \approx \mathrm{Tr}\left(F^{-1}\left(\hat{z}\right)\right)+\sum_{|\beta |=1}^{2}{\left.\frac{1}{\beta !}\frac{\partial^{\left|\beta \right|}\mathrm{Tr}\left(F^{-1}\left(z\right)\right)}{\partial z^{\beta }}\right|}_{\hat{z}}{\left(z-\hat{z}\right)}^{\beta }\\ \; & =\mathrm{Tr}\left(F^{-1}\left(\hat{z}\right)\right)+\left[\frac{\partial \mathrm{Tr}(F^{-1}\left(z\right))}{\partial x}\Delta x+\frac{\partial \mathrm{Tr}(F^{-1}\left(z\right))}{\partial y}\Delta y\right]{|}_{\hat{z}}\\ \; & \;+\frac{1}{2}\left[\frac{\partial^{2}\mathrm{Tr}\left(F^{-1}\left(z\right)\right)}{\partial x^{2}}\Delta x^{2}+2\frac{\partial^{2}\mathrm{Tr}\left(F^{-1}\left(z\right)\right)}{\partial x\partial y}\Delta x\Delta y+\frac{\partial^{2}\mathrm{Tr}\left(F^{-1}\left(z\right)\right)}{\partial y^{2}}\Delta y^{2}\right]{|}_{\hat{z}} \end{array}$$

Let $h\left(z\right)=\mathrm{Tr}(F^{-1}\left(z\right))$ and $\Delta z=z-\hat{z}={\left[\Delta x,\Delta y\right]}^{T}$. The second-order expansion in Equation (62) can also be expressed in the compact quadratic form

(63) $$h\left(\hat{z}+\Delta z\right)\approx h\left(\hat{z}\right)+\nabla h{\left(\hat{z}\right)}^{T}\Delta z+\frac{1}{2}\Delta z^{T}H\left(\hat{z}\right)\Delta z$$

where $H(\hat{z})$ is the Hessian matrix of h(z) evaluated at the estimated target position. Taking the mathematical expectation of Equation (63), the first-order term becomes $E\left[\nabla h{\left(\hat{z}\right)}^{T}\Delta z\right]=\nabla h{\left(\hat{z}\right)}^{T}E\left[\Delta z\right]$. Under the symmetric zero-mean Gaussian uncertainty model, this first-order term equals zero. The remaining second-order term is determined by the second-order moment $E[\Delta z\Delta z^{T}]$, which is represented by the covariance matrix M in the subsequent derivation. Obviously, evaluating the mathematical expectation of the second-order Taylor expansion is significantly more computationally tractable than directly calculating the complex integrations in Equation (51) over the uncertainty region Φ, weighted by the real-time multivariate Gaussian distribution $f(\hat{z};z)$. In the two-dimensional spatial deviation vector, Δx represents the instantaneous distance between the true and estimated target positions along the x-axis, and Δy represents the corresponding distance along the y-axis.

The mathematical expectation expression of the second-order Taylor expansion in Equation (62) is then given as follows

(64) $$\begin{array}{cc} E\left[\mathrm{Tr}(F^{-1}\left(z\right))\right]\approx \; & E\left[\mathrm{Tr}\left(F^{-1}\left(\hat{z}\right)\right)\right]+\left[\frac{\partial \mathrm{Tr}(F^{-1}\left(z\right))}{\partial x}E\left[\Delta x\right]+\frac{\partial \mathrm{Tr}(F^{-1}\left(z\right))}{\partial y}E\left[\Delta y\right]\right]{|}_{\hat{z}}\\ \; & +\frac{1}{2}[\frac{\partial^{2}\mathrm{Tr}\left(F^{-1}\left(z\right)\right)}{\partial x^{2}}E\left[\Delta x^{2}\right]+2\frac{\partial^{2}\mathrm{Tr}\left(F^{-1}\left(z\right)\right)}{\partial x\partial y}E\left[\Delta x\Delta y\right]\\ \; & \;\;+\frac{\partial^{2}\mathrm{Tr}\left(F^{-1}\left(z\right)\right)}{\partial y^{2}}E\left[\Delta y^{2}\right]]{|}_{\hat{z}} \end{array}$$

where E[·] denotes the mathematical expectation. To calculate Equation (64), the statistical characteristics must be analyzed item by item. Firstly, the zero-order term (the first term in Equation (64)) is evaluated at a fixed Taylor expansion center, acting as a constant value independent of the uncertain area Φ. Thus, the expectation of the zero-order term is the function value itself: $E\left[\mathrm{Tr}\left(F^{-1}\left(\hat{z}\right)\right)\right]=\mathrm{Tr}\left(F^{-1}\left(\hat{z}\right)\right)$. Secondly, the first-order terms (the second terms in Equation (64)) are driven by the expectations of the spatial deviations (E[Δx] and E[Δy]). Since the target-position uncertainty follows a symmetric zero-mean Gaussian distribution $f(\hat{z};z)$, the expectations of the odd-order terms are all equal to zero, i.e., E[Δx]=0 and E[Δy]=0. Therefore, the entire first-order correlative terms can be canceled out. Thirdly, to obtain the second-order terms (the third terms in Equation (64)), the second-order spatial expectations $E[\Delta x^{2}]$, $E[\Delta y^{2}]$, and the cross-term E[ΔxΔy] must be determined, which are strictly defined by the variance and covariance elements of the target position uncertainty matrix M. M represents the real-time covariance matrix of the target’s position uncertainty, and $\sigma_{x}^{2}$ and $\sigma_{y}^{2}$ denote the error variances in the x-axis and y-axis, respectively, and $\sigma_{xy}$ is the cross-axis covariance in M

(65) $$\left[\begin{array}{cc} E[\Delta x^{2}] & E[\Delta x\Delta y]\\ E[\Delta x\Delta y] & E[\Delta y^{2}] \end{array}\right]\approx M=\left[\begin{array}{cc} \sigma_{x}^{2} & \sigma_{xy}\\ \sigma_{xy} & \sigma_{y}^{2} \end{array}\right]$$

By substituting the zero-order term, first-order terms, second-order terms, and Equation (65) into Equation (64), we can obtain the following equation as

(66) $$\begin{array}{cc} E[\mathrm{Tr}\left(F^{-1}\left(z\right)\right)]= & \mathrm{Tr}(F^{-1}\left(\hat{z}\right))\\ \; & +\frac{1}{2}\left[\sigma_{x}^{2}\frac{\partial^{2}\mathrm{Tr}\left(F^{-1}\left(z\right)\right)}{\partial x^{2}}+\sigma_{y}^{2}\frac{\partial^{2}\mathrm{Tr}\left(F^{-1}\left(z\right)\right)}{\partial y^{2}}+2\sigma_{xy}\frac{\partial^{2}\mathrm{Tr}\left(F^{-1}\left(z\right)\right)}{\partial x\partial y}\right]{|}_{\hat{z}} \end{array}$$

Although Equation (66) successfully transforms numerical integration into an analytical form, directly calculating the second-order partial derivatives of an inverse matrix trace (e.g., ${\left.\frac{\partial^{2}\mathrm{Tr}\left(F^{-1}\left(z\right)\right)}{\partial x^{2}}\right|}_{\hat{z}}$) is extremely difficult. Therefore, the matrix perturbation theory is introduced here to transform the entire calculation into basic algebraic operations. The derivative identities used below follow from $dF^{-1}=-F^{-1}\left(dF\right)F^{-1}$ and standard matrix differential calculus [34,35]. For two spatial variables p,q∈{x,y}, let $F_{,p}=\partial F/\partial p$ and $F_{,pq}=\partial^{2}F/\partial p\partial q$. With $h\left(z\right)=\mathrm{Tr}(F^{-1}\left(z\right))$ and $C=F^{-1}\left(\hat{z}\right)$, the second derivative of the inverse-matrix trace can be written as

(67) $$\frac{\partial^{2}h}{\partial p\partial q}=\mathrm{Tr}\left(CF_{,q}CF_{,p}C+CF_{,p}CF_{,q}C-CF_{,pq}C\right).$$

where all derivatives are evaluated at the expansion center $\hat{z}$. Using this relation together with the symmetry of the FIM terms gives the following compact derivative expressions. At the expansion center, we define the first-order partial derivatives of the FIM shown in Equation (48) with respect to the explicit spatial x-axis and y-axis as $F_{,x}={\left.\frac{\partial F(z)}{\partial x}\right|}_{\hat{z}}$ and $F_{,y}={\left.\frac{\partial F(z)}{\partial y}\right|}_{\hat{z}}$. Similarly, the second-order partial derivatives can be denoted as $F_{,xx}={\left.\frac{\partial^{2}F\left(z\right)}{\partial x^{2}}\right|}_{\hat{z}}$, $F_{,yy}={\left.\frac{\partial^{2}F\left(z\right)}{\partial y^{2}}\right|}_{\hat{z}}$, and $F_{,xy}={\left.\frac{\partial^{2}F\left(z\right)}{\partial x\partial y}\right|}_{\hat{z}}$. By leveraging the fundamental matrix partial derivative identities $\frac{\partial (F^{-1}\left(z\right))}{\partial x}=-F^{-1}\left(z\right)\frac{\partial F(z)}{\partial x}F^{-1}\left(z\right)$ and $\frac{\partial (F^{-1}\left(z\right))}{\partial y}=-F^{-1}\left(z\right)\frac{\partial F(z)}{\partial y}F^{-1}\left(z\right)$, the analytical formulations for these required first- and second-order partial derivatives can be rigorously derived in a step-by-step manner. Therefore, the partial derivatives of the first-order Taylor expansion can be written as

(68) $${\left.\frac{\partial \mathrm{Tr}(F^{-1}\left(z\right))}{\partial x}\right|}_{\hat{z}}=-\mathrm{Tr}\left(CF_{,x}C\right)$$

(69) $${\left.\frac{\partial \mathrm{Tr}(F^{-1}\left(z\right))}{\partial y}\right|}_{\hat{z}}=-\mathrm{Tr}\left(CF_{,y}C\right)$$

Based on the derived first-order terms, the partial derivatives of the second-order Taylor expansions can be subsequently obtained by iteratively applying the differential chain rule, yielding

(70) $${\left.\frac{\partial^{2}\mathrm{Tr}\left(F^{-1}\left(z\right)\right)}{\partial x^{2}}\right|}_{\hat{z}}=\mathrm{Tr}\left(C(2F_{,x}CF_{,x}-F_{,xx})C\right)$$

(71) $${\left.\frac{\partial^{2}\mathrm{Tr}\left(F^{-1}\left(z\right)\right)}{\partial y^{2}}\right|}_{\hat{z}}=\mathrm{Tr}\left(C(2F_{,y}CF_{,y}-F_{,yy})C\right)$$

(72) $${\left.\frac{\partial^{2}\mathrm{Tr}\left(F^{-1}\left(z\right)\right)}{\partial x\partial y}\right|}_{\hat{z}}=\mathrm{Tr}\left(C(2F_{,x}CF_{,y}-F_{,xy})C\right)$$

Let $H(\hat{z})$ denote the 2×2 Hessian matrix which can be described as follows

(73) $$H\left(\hat{z}\right)=\left[\begin{array}{cc} {\left.\frac{\partial^{2}\mathrm{Tr}\left(F^{-1}\left(z\right)\right)}{\partial x^{2}}\right|}_{\hat{z}} & {\left.\frac{\partial^{2}\mathrm{Tr}\left(F^{-1}\left(z\right)\right)}{\partial x\partial y}\right|}_{\hat{z}}\\ {\left.\frac{\partial^{2}\mathrm{Tr}\left(F^{-1}\left(z\right)\right)}{\partial x\partial y}\right|}_{\hat{z}} & {\left.\frac{\partial^{2}\mathrm{Tr}\left(F^{-1}\left(z\right)\right)}{\partial y^{2}}\right|}_{\hat{z}} \end{array}\right]$$

By substituting Equations (65)–(73) into Equation (51), the final closed-form analytical expression for the trace of the global CRLB is obtained as

(74) $$\mathrm{Tr}\left({\mathrm{CRLB}}_{\mathrm{analytical}}\right)=\mathrm{Tr}\left(F^{-1}\left(\hat{z}\right)\right)+\frac{1}{2}\mathrm{Tr}\left(MH(\hat{z})\right)$$

After obtaining the analytical expression of the global CRLB trace, the UAV path optimization mathematical model in Equation (59) can be formally updated to

(75) $$\begin{array}{cc} \; & \min_{w_{i}^{t+1}}\arg\;\mathrm{Tr}\left({\mathrm{CRLB}}_{\mathrm{analytical}}^{t+1}\right)\\ \; & \mathrm{subject}\mathrm{to}\left\{\begin{array}{c} R_{i}^{t}>R_{\min}\\ D_{i}^{t}>D_{\min}\\ L_{i}^{t}>L_{\min}\\ \phi_{i}^{t}\in \left(\alpha_{i}^{t}-\Delta \alpha_{\mathrm{target}},\;\alpha_{i}^{t}+\Delta \alpha_{\mathrm{target}}\right) \end{array}\right.\;i=0,1,2,\ldots ,I-1 \end{array}$$

### 4.3. PSO Algorithm

To efficiently and accurately search for the optimal trajectory for the UAVs, minimizing the objective function in Equation (75), the PSO algorithm is deployed in this paper.

Suppose there are *N* particles searching in each iteration, and each particle *j* (1≤j≤N) has information about its own historical best position $p^{j}$ and its current position $\Theta^{j}={\left[\phi_{0}^{j},\phi_{1}^{j},\ldots ,\phi_{I-1}^{j}\right]}^{T}$, where $\phi_{i}^{j}\left(i=0,1,2,\ldots ,I-1\right)$ denotes the deflection angle of the *i*th UAV for the *j*th particle, and *I* is the number of UAVs. The velocity of the *j*th particle is $u^{j}$, and the global best particle is denoted as $p^{g}$. The velocity and the position of the particle are updated as follows, where *k* denotes the iteration step

(76) $$\begin{array}{cc} u^{j}\left(k+1\right) & =\xi \left(k\right)u^{j}\left(k\right)+c_{1}\left(k\right)r_{1}\left(k\right)\left(p^{j}\left(k\right)-\Theta^{j}\left(k\right)\right)\\ \; & +c_{2}\left(k\right)r_{2}\left(k\right)\left(p^{g}\left(k\right)-\Theta^{j}\left(k\right)\right), \end{array}$$

(77) $$\Theta^{j}\left(k+1\right)=\Theta^{j}\left(k\right)+u^{j}\left(k+1\right)$$

where ξ(k) is the inertia weight, which decreases with iteration time as $\xi \left(k\right)=0.9-0.5\left(k/k_{\max}\right)$. $k_{\max}$ is the maximum number of iterations. $c_{1}\left(k\right)$ and $c_{2}\left(k\right)$ are individual learning and social learning factors, which vary with iteration time as $c_{1}\left(k\right)=2.5-1.5\left(k/k_{\max}\right)$ and $c_{2}\left(k\right)=0.5+1.5\left(k/k_{\max}\right)$, respectively. $r_{1}\left(k\right)$ and $r_{2}\left(k\right)$ are random independent variables. The velocity of the particle is limited by a maximum velocity $v_{\max}\left(k\right)$, which prevents the particles from leaping disproportionately and avoids unreasonable movements within the solution space.

The current position of each particle represents one solution for the UAVs’ trajectory. The performance of each particle is evaluated via a fitness function F(.). To clarify the treatment of infeasible particles, the objective term and the constraint penalty term are written together as

(78) $$F^{j}\left(k\right)=F\left(\Theta^{j}\left(k\right)\right)=J_{\mathrm{CRLB}}\left(\Theta^{j}\left(k\right)\right)+J_{\mathrm{penalty}}\left(\Theta^{j}\left(k\right)\right)$$

where $J_{\mathrm{CRLB}}$ can be selected as the single-point CRLB, Monte Carlo global CRLB, or analytical global CRLB objective in the compared simulations. The penalty term $J_{\mathrm{penalty}}$ increases when the minimum turning radius, minimum spacing distance, minimum approach distance, or deflection-angle constraint is violated. $\Theta^{j}\left(k\right)$ denotes the current position of the *j*th particle, $F^{j}\left(k\right)$ represents its corresponding scalar fitness value, and a lower fitness value indicates a better feasible UAV trajectory candidate. Consequently, the historical best position of the individual particle $p^{j}\left(k\right)$ can be updated as

(79) $$p^{j}\left(k+1\right)=\left\{\begin{array}{cc} \Theta^{j}\left(k+1\right), & \mathrm{if}\;F^{j}\left(k+1\right)<F\left(p^{j}\left(k\right)\right)\\ p^{j}\left(k\right), & \mathrm{if}\;F^{j}\left(k+1\right)\geq F\left(p^{j}\left(k\right)\right) \end{array}\right.,$$

and the global best position $p^{g}\left(k\right)$ found by all particles is updated by identifying the minimum fitness value among all particles’ historical bests

(80) $$p^{g}\left(k+1\right)=\arg\min_{1\leq j\leq N}F\left(p^{j}\left(k+1\right)\right)$$

When either the improvement of the global best value over five consecutive generations is less than a predefined threshold, or the maximum number of iterations $k=k_{\max}$ is reached, the iteration will stop and output the optimization result; thus, the optimal UAV path optimization will be obtained. Here, a general procedure of the path optimization PSO algorithm is given in Algorithm 1.
**Algorithm 1:** The PSO Algorithm for UAV Cooperative Path Optimization 

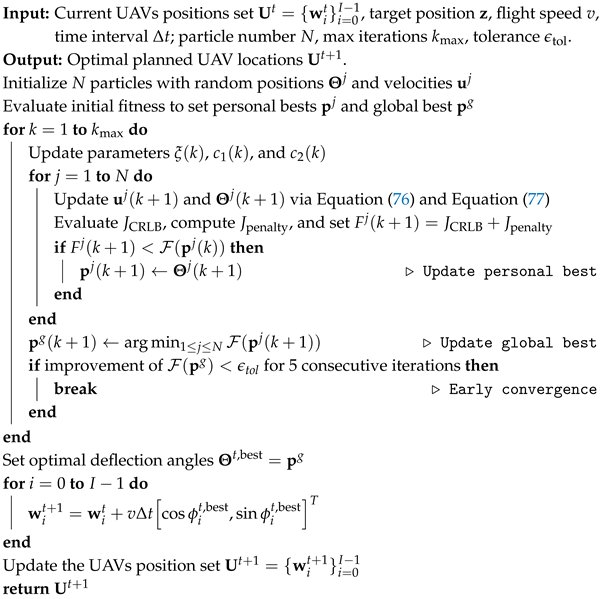



## 5. Numerical Results

### 5.1. Simulation Parameter Set and Initial Topology Set

**Figure 3:**
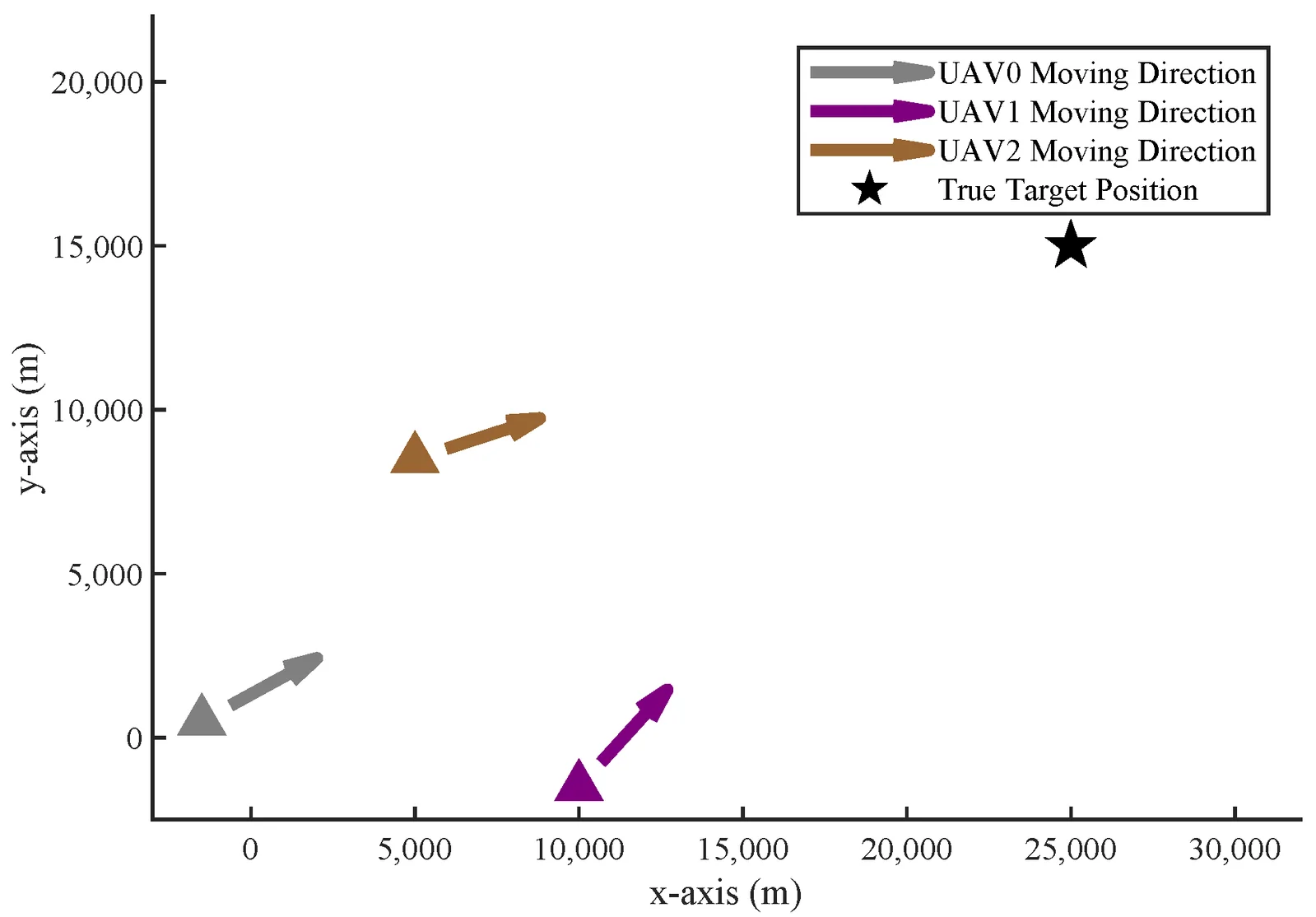
Initial topology diagram of UAVs. Triangles indicate the initial UAV positions, arrows indicate the corresponding moving directions, and the star indicates the true target position.

In this paper, three UAVs (I=3) and one target are considered, with the UAVs moving toward the target. The initial positions of the UAVs and the target are set as follows: UAV0 is located at [−1500,500] m, UAV1 at [10,000,−1500] m, UAV2 at [5000,8500] m, and the target is located at [25,000,15,000] m. The speed of the UAVs is set to v=50 m/s, the planning interval is Δt=10 s, and the corresponding movement radius per step is 500 m. The initial topology is shown in Figure 3. The simulations use the Chan localization algorithm described in Section 2. All numerical simulations were conducted in MATLAB (R2024a, The MathWorks, Inc., Natick, MA, USA). No physical equipment was used in this study.

In this part, we compare the simulation results obtained by different path optimization algorithms, including: (1) the path optimization algorithm based on the single-point CRLB (single-point method for short); (2) the path optimization algorithm based on the global CRLB calculated by the Monte Carlo method (MC method for short); and (3) the path optimization algorithm based on the global CRLB calculated by the analytical method (analytical method for short). The optimized UAVs’ path, the root mean square error (RMSE) results, the CRLB results, and the computation efficiency are shown and compared as follows. The main simulation parameters are listed in Table 1. The outer Monte Carlo number for the reported mean curves and confidence intervals is M=200. The MC global CRLB objective also uses 200 target-position samples, and each RMSE point is evaluated with R=30 inner TDOA-noise resamples. The displayed CRLB metric is evaluated at the true target position, while the global CRLB is used as the uncertainty-aware planning objective and for validating the analytical approximation.

### 5.2. Simulation Results of the Optimized UAVs’ Paths

**Figure 4:**
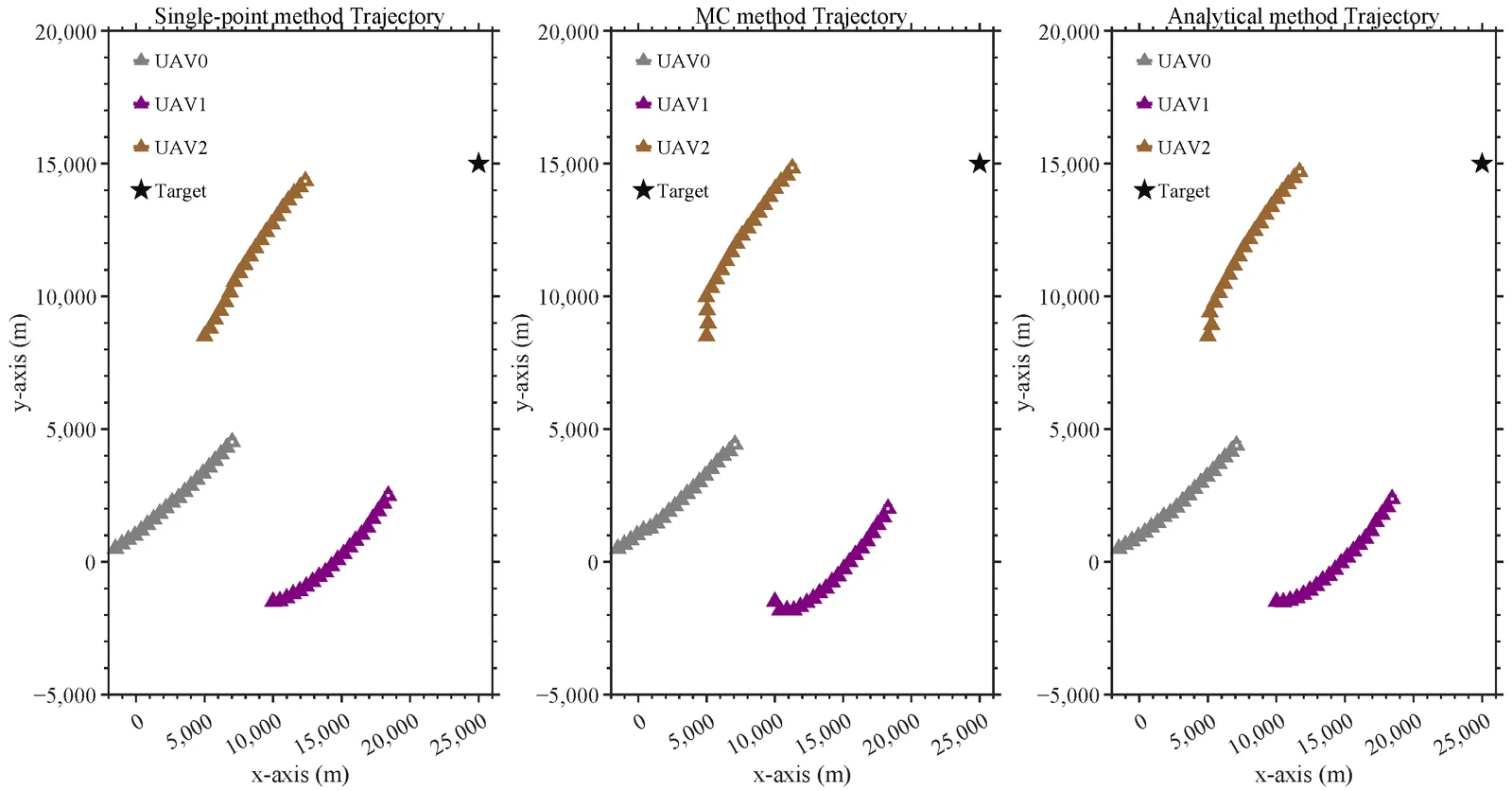
UAVs’ trajectory results optimized by the single-point method, MC method, and analytical method. The coordinate labels in the figure use comma-separated thousands and a mathematical minus sign.

Figure 4 shows the optimized UAVs’ trajectory results obtained by the three-path optimization algorithms. The single-point method optimizes the UAVs’ geometry only around the current target estimate. In contrast, the MC method and the analytical method account for the target-position uncertainty distribution during planning, leading to more uncertainty-aware observation geometries.

### 5.3. RMSE and CRLB Comparison Based on Different Optimization Algorithms

To evaluate the localization performance by different UAV path optimization algorithms, RMSE is used as a metric. For clarity, the RMSE evaluation uses two sampling levels. The outer Monte Carlo index is m=1,…,M, where M=200 independent simulation runs are used to report the mean curves and 95% confidence intervals. For each outer run and each time step, R=30 inner TDOA-noise resamples are generated to estimate the conditional localization error under the planned UAVs’ geometry. Let $\left({\hat{x}}_{m,r}\left(t\right),{\hat{y}}_{m,r}\left(t\right)\right)$ be the Chan localization result obtained from the *r*th inner TDOA-noise resample in the *m*th outer run at time step *t*. The run-level RMSE is computed as

(81) $${\mathrm{RMSE}}_{m}\left(t\right)={\left[\frac{1}{R}\sum_{r=1}^{R}\left({\left({\hat{x}}_{m,r}\left(t\right)-x\right)}^{2}+{\left({\hat{y}}_{m,r}\left(t\right)-y\right)}^{2}\right)\right]}^{1/2}.$$

The reported RMSE curve is then averaged over the outer runs,

(82) $$\bar{\mathrm{RMSE}}\left(t\right)=\frac{1}{M}\sum_{m=1}^{M}{\mathrm{RMSE}}_{m}\left(t\right),$$

and the 95% confidence interval is computed from the *M* outer-run RMSE values. Therefore, M=200 and R=30 refer to different sampling levels and are not conflicting settings. The plotted curves are not processed by a monotone envelope.

**Figure 5:**
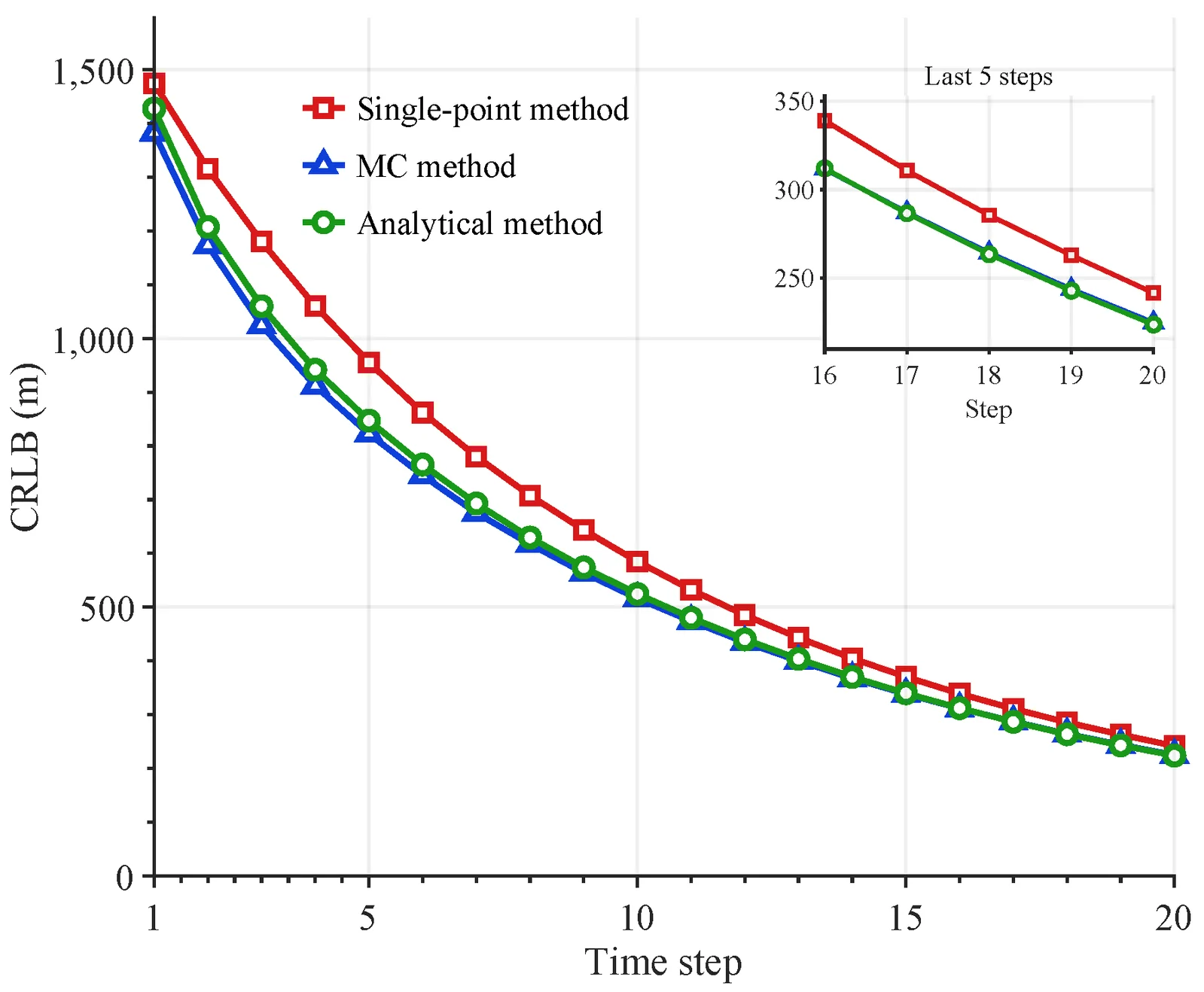
CRLB curve comparison between three UAV path optimization algorithms.

**Figure 6:**
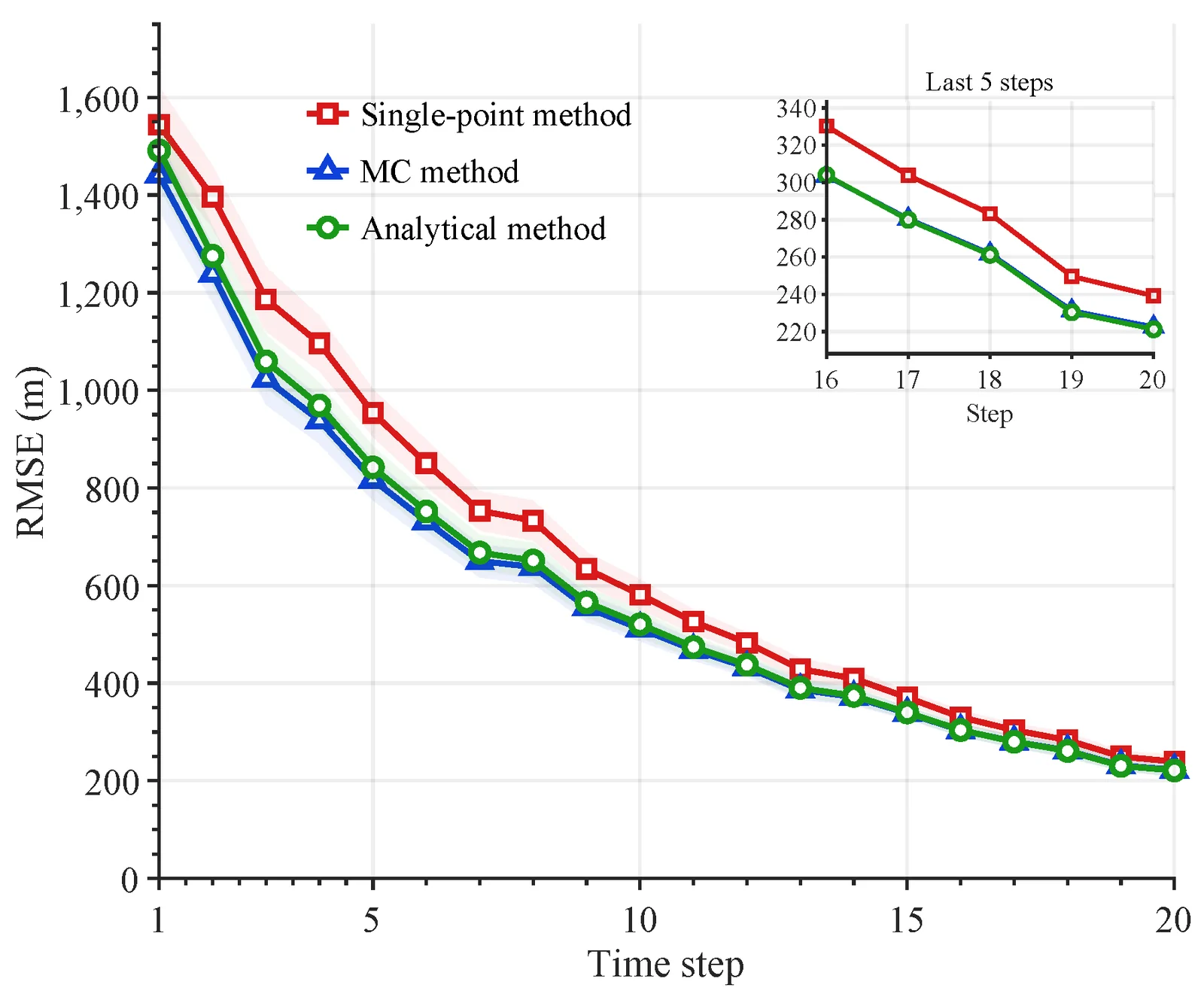
RMSE curve comparison between three UAV path optimization algorithms.

The CRLB and RMSE curves are shown in Figure 5 and Figure 6, respectively. Both global CRLB methods reduce the CRLB and localization error relative to the single-point method. The analytical method closely follows the MC method while avoiding repeated numerical integration during each objective evaluation.

Table 2 summarizes the raw time-indexed numerical results. At the terminal step, the single-point method gives a CRLB of 241.62 m and an RMSE of 239.20 m. The MC method reduces these values to 224.80 m and 222.49 m, respectively, while the analytical method gives 223.72 m and 221.24 m. No localization failures or outliers are observed for the three methods in the M=200 runs.

To further quantitatively analyze the RMSE performance difference between the three UAV path optimization algorithms, the RMSE data by different algorithms are given and compared in Table 2. We define that

(83) $$RMSE_{\mathrm{Imp}}=\left(RMSE_{\mathrm{single}}-RMSE_{\mathrm{global}}\right)/RMSE_{\mathrm{single}}\times 100\%,$$

where $RMSE_{\mathrm{Imp}}$ represents the performance improvement percentage between the single-point method and the global CRLB method, $RMSE_{\mathrm{single}}$ represents the RMSE by the single-point method, and $RMSE_{\mathrm{global}}$ represents the RMSE by the global CRLB method.

Similarly, the CRLB improvement percentage is defined as

(84) $$CRLB_{\mathrm{Imp}}=\left(CRLB_{\mathrm{single}}-CRLB_{\mathrm{global}}\right)/CRLB_{\mathrm{single}}\times 100\%,$$

where $CRLB_{\mathrm{Imp}}$ represents the performance improvement percentage between the single-point method and the global CRLB method. Using the single-point method as the baseline, the MC method achieves an average CRLB improvement of 10.41% and a final CRLB improvement of 6.96%. The analytical method achieves an average CRLB improvement of 9.11% and a final CRLB improvement of 7.41%. The corresponding average RMSE improvements are 10.62% for the MC method and 9.27% for the analytical method. These results indicate that the uncertainty-aware objectives provide better observation geometry than the single-point method, and that the analytical approximation preserves the main localization-performance benefit of the MC method.

### 5.4. Optimizer and Baseline Comparison

**Figure 7:**
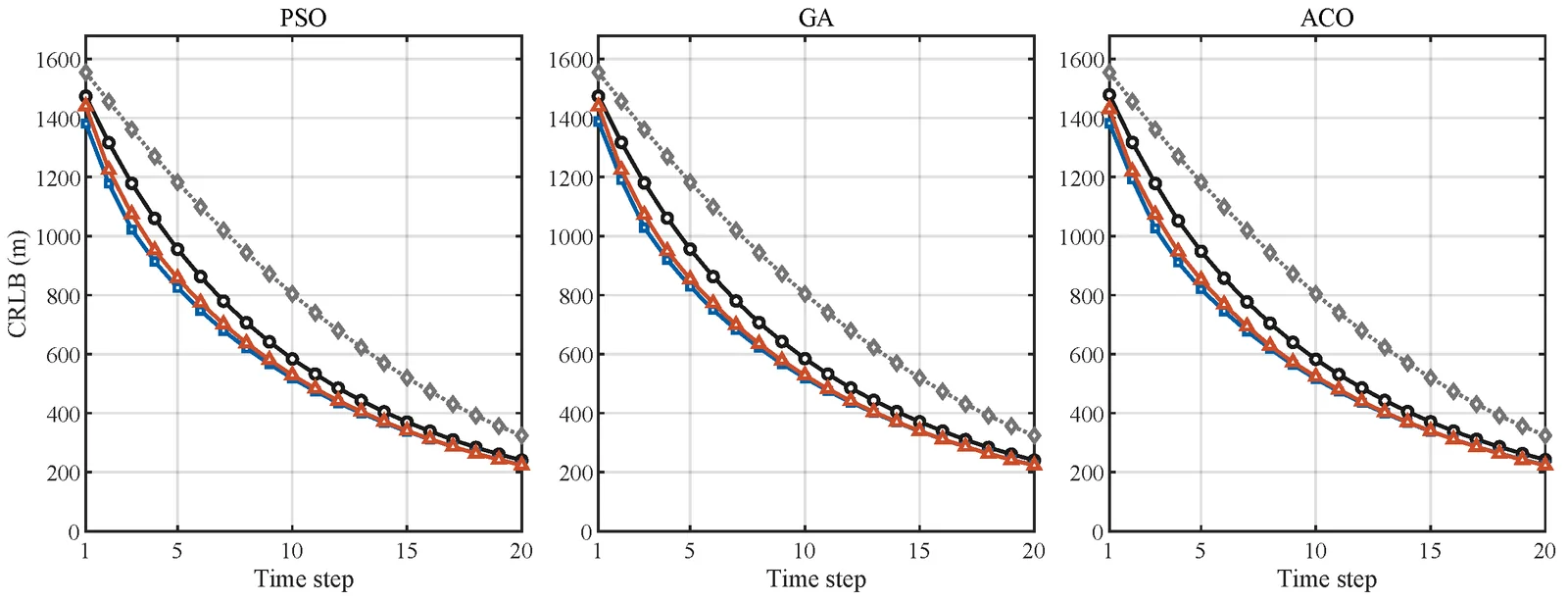
Three-UAV optimizer comparison using CRLB.

**Figure 8:**
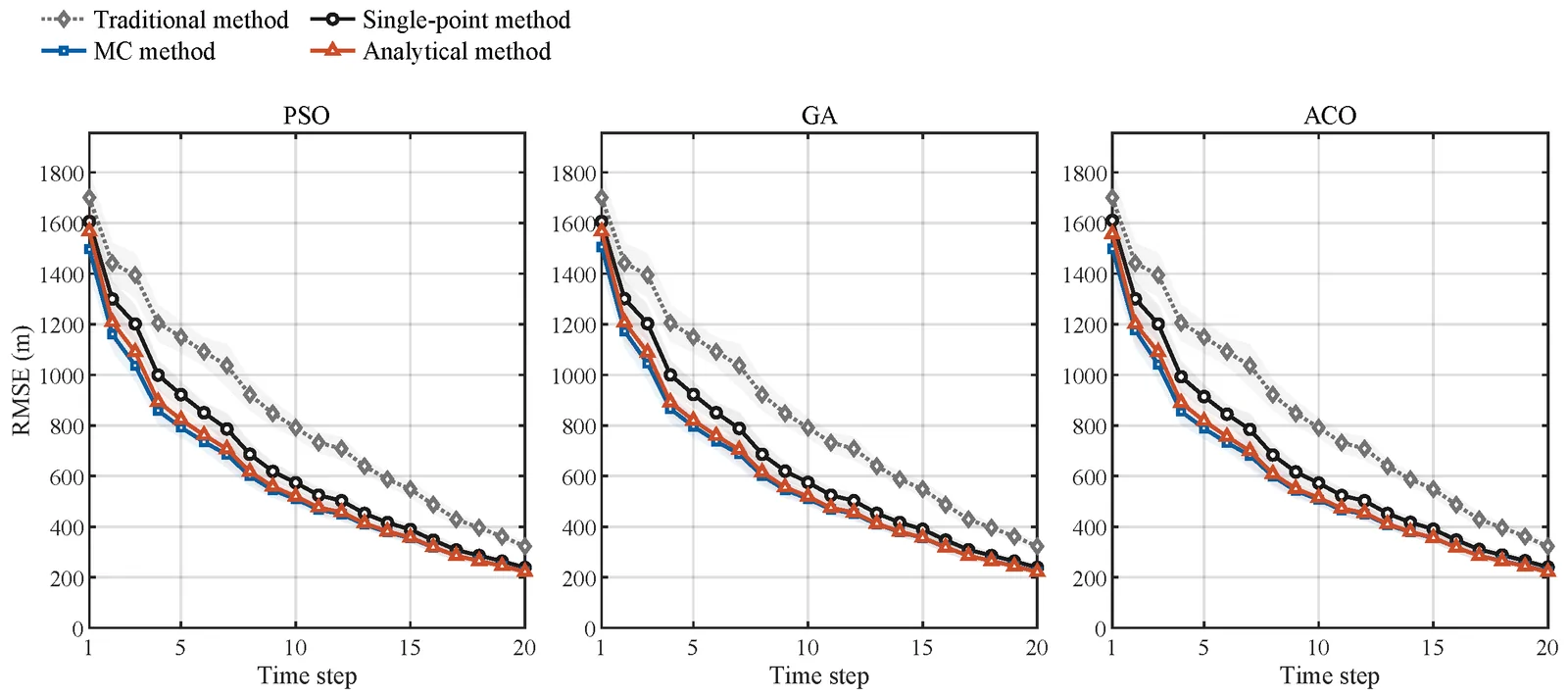
Three-UAV optimizer comparison using RMSE.

To test whether the improvement is caused by a favorable PSO setting rather than by the proposed uncertainty-aware objective, an additional three-UAV optimizer comparison was conducted under the same Chan localization setting used in the main experiment. The same four planning choices were evaluated with PSO, GA, and ACO: the traditional method, single-point method, MC method, and analytical method. The traditional method denotes the fixed-formation flight group, whereas the other three methods use the corresponding CRLB-based objectives. This comparison uses M=200, 200 target-position samples for the MC global CRLB objective, 30 inner TDOA-noise resamples for RMSE evaluation, and v=50m/s. The solver settings are listed in Table 3, and the corresponding CRLB and RMSE curves are shown in Figure 7 and Figure 8.

For PSO, the terminal CRLB values of the traditional method, single-point method, MC method, and analytical method are 324.12 m, 240.26 m, 224.11 m, and 223.29 m, respectively. The corresponding terminal RMSE values are 322.59 m, 238.58 m, 222.52 m, and 221.76 m. For GA, the terminal CRLB values are 324.12 m, 240.43 m, 223.63 m, and 222.56 m, and the terminal RMSE values are 322.59 m, 238.91 m, 222.15 m, and 221.05 m. For ACO, the terminal CRLB values are 324.12 m, 242.07 m, 224.00 m, and 222.75 m, and the terminal RMSE values are 322.59 m, 240.16 m, 222.59 m, and 221.12 m. These results indicate that the improvement of the global CRLB objectives is not an artifact of PSO hyperparameter tuning; rather, the uncertainty-aware objective provides consistent localization-performance gains across the three optimizers.

In the present controlled experiment, the fixed-formation-to-single-point reductions are 25.87% in terminal CRLB and 26.05% in terminal RMSE, and the additional single-point-to-analytical reductions are 7.06% and 7.05%, respectively; equivalently, the analytical method reduces terminal CRLB and RMSE by 31.11% and 31.26% relative to the fixed-formation baseline.

### 5.5. Analytical Approximation Accuracy and Computational Cost

**Figure 9:**
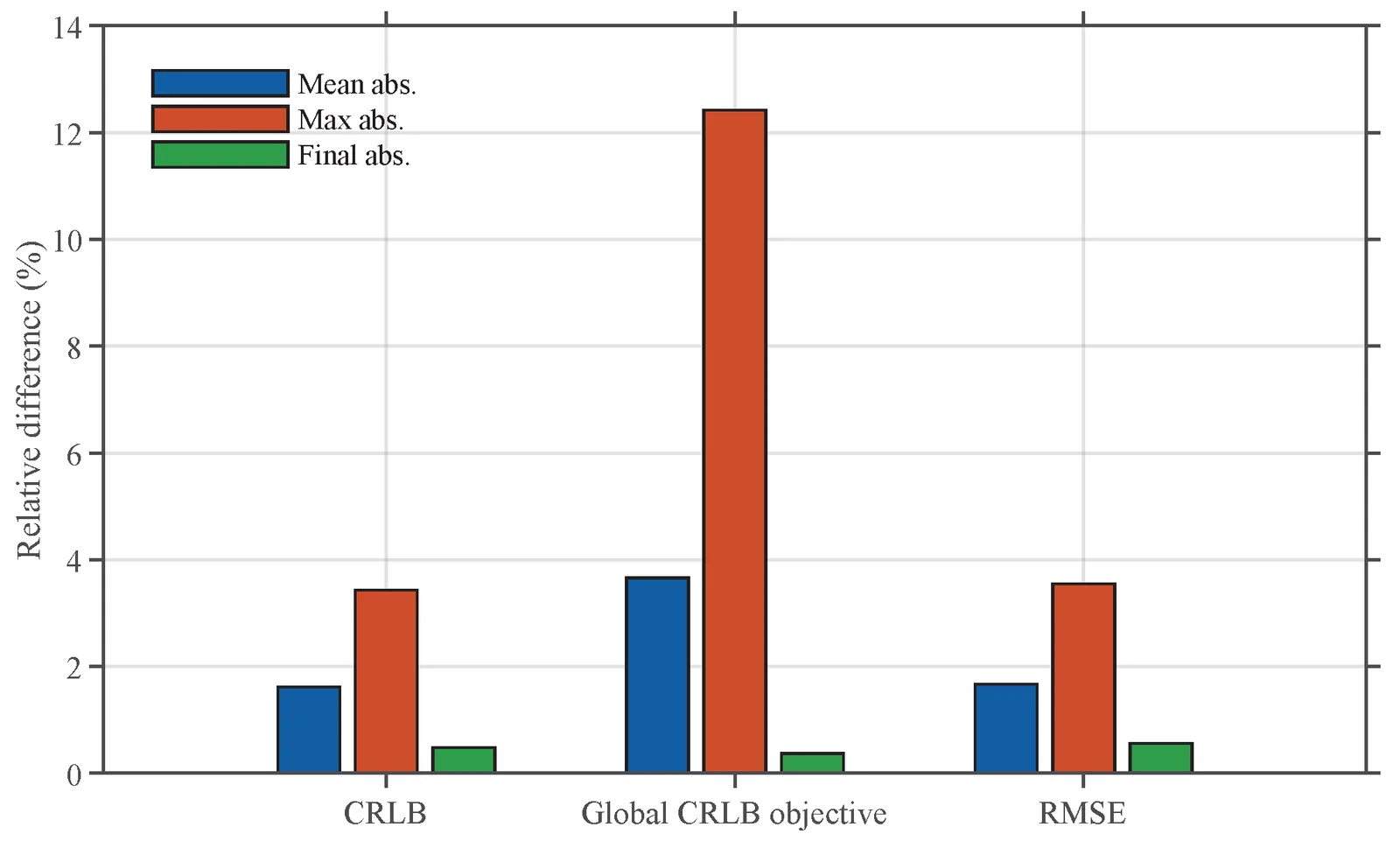
Analytical-vs-MC relative differences.

Table 4 compares the analytical method with the MC method, and Figure 9 presents the corresponding relative differences. The mean absolute relative error between the analytical method and MC method is 1.62% for CRLB and 1.67% for RMSE. The final relative error is −0.48% for CRLB and −0.56% for RMSE, showing that the analytical objective remains close to the MC reference in the final planning stage.

**Figure 10:**
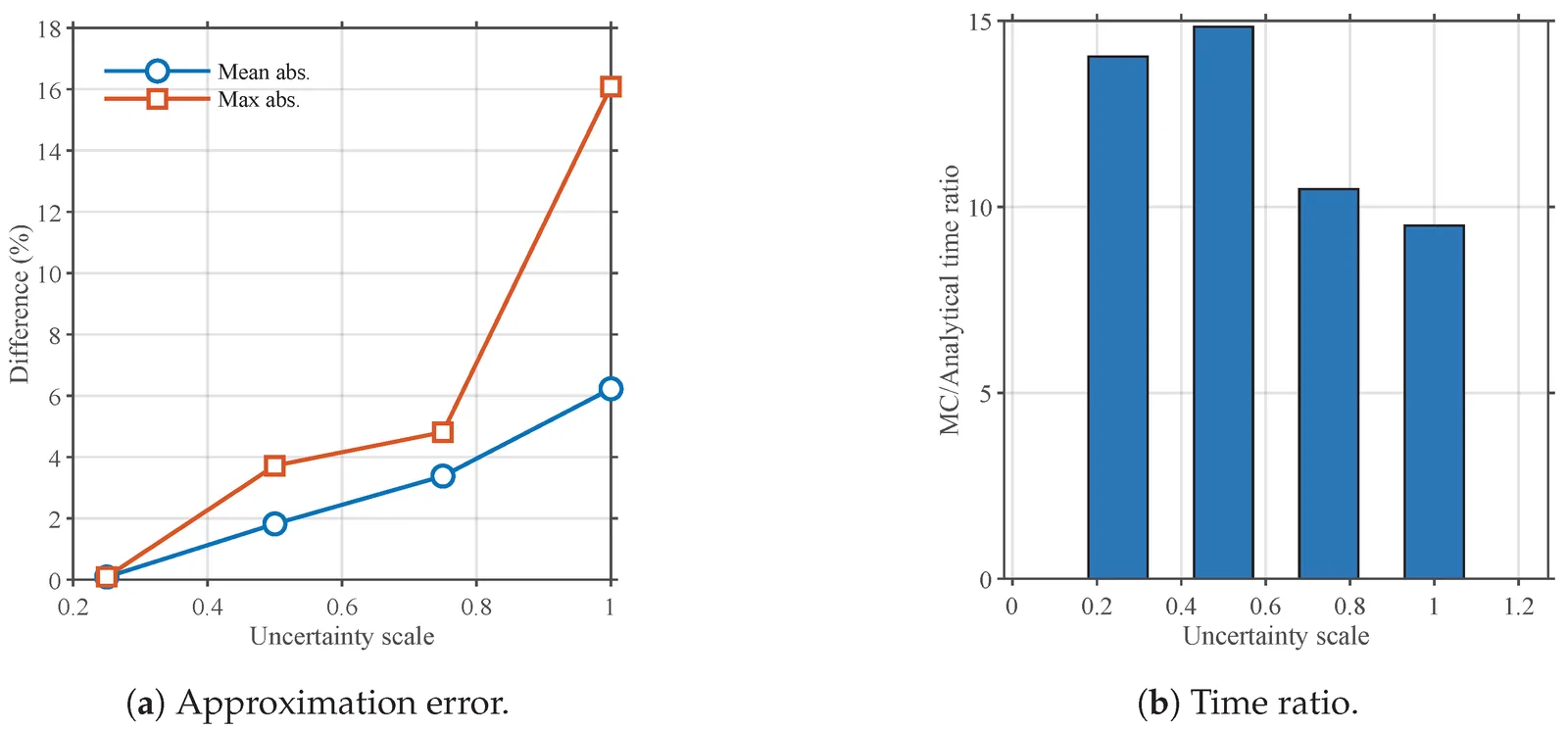
Uncertainty-scale effect on approximation accuracy and timing.

To further examine the validity range of the second-order Taylor approximation, a fixed-geometry target-position uncertainty-scale analysis was conducted. This analysis scales the target-position uncertainty covariance used in the global CRLB calculation and is therefore different from the measurement-noise sensitivity test reported later. As shown in Table 5 and Figure 10, the analytical global CRLB closely follows the MC global CRLB for small to moderate uncertainty scales. The approximation error in Figure 10a and the time ratio in Figure 10b jointly show the accuracy–efficiency tradeoff. The mean absolute analytical-vs-MC difference is below 3.38% for uncertainty scales up to 0.75, while the analytical evaluation remains about one order of magnitude faster than the MC reference. At the broader uncertainty scale of 1.00, the mean difference increases to 6.23%, indicating the expected degradation of a local second-order approximation when the uncertainty support becomes wider. Therefore, the analytical approximation is used as an efficient surrogate under the adopted uncertainty setting, while broad-uncertainty cases are interpreted as stress cases rather than as the primary accuracy range.

### 5.6. Robustness to Increasing Target-Position Error

**Figure 11:**
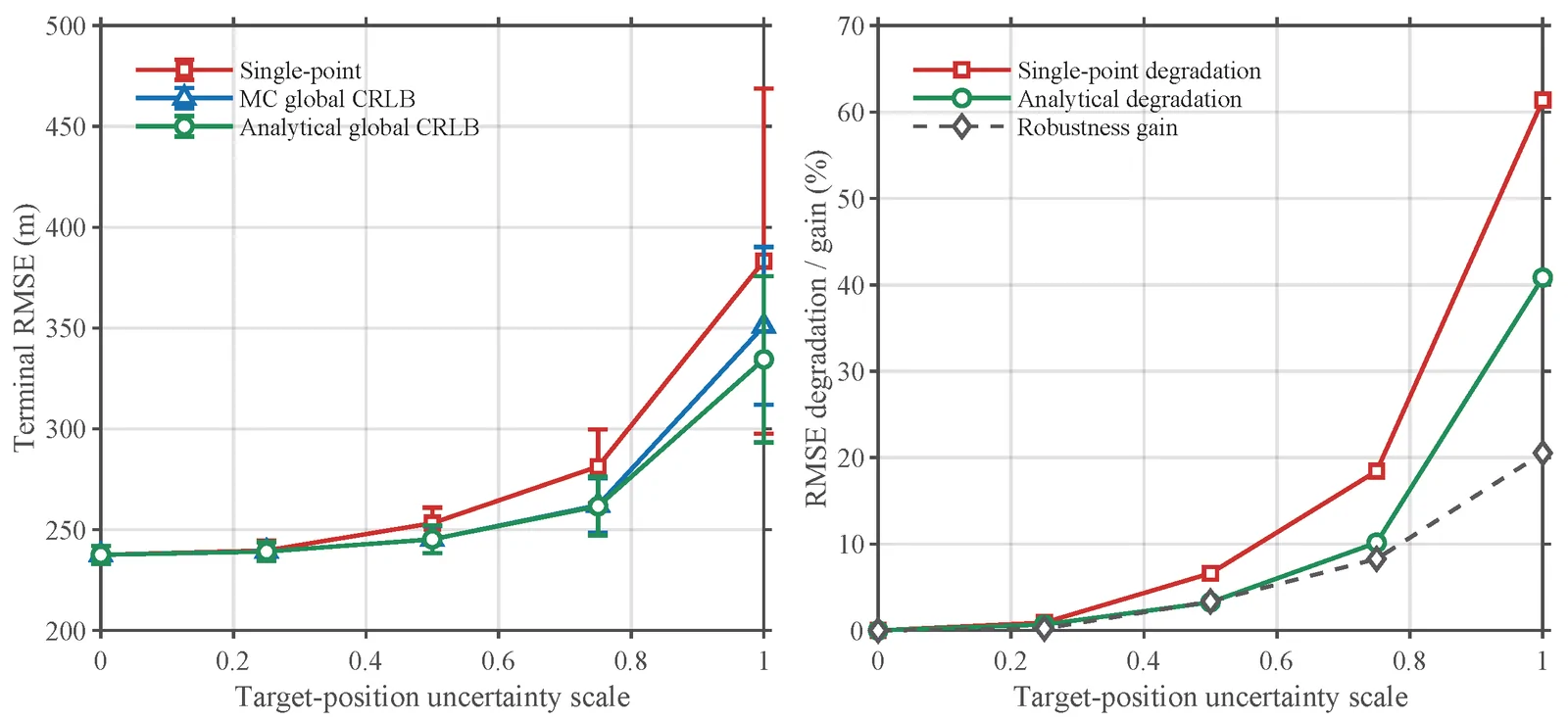
Terminal RMSE and its relative degradation as the target-position uncertainty scale increases.

To examine robustness to target-position error, the true target position was held fixed. The planner-side error was scaled as $e\left(s\right)=se_{0}$, and its covariance was scaled as **∑***_z_* $\left(s\right)=s^{2}$**∑***_z_*, where s∈{0,0.25,0.50,0.75,1.00}. At each scale, the single-point, MC global-CRLB, and analytical global-CRLB objectives were optimized under the same motion constraints and the same controlled PSO budget. The additional sweep used M=200 outer runs, R=30 inner TDOA-noise resamples for each RMSE point, and 200 target-position samples for the MC global-CRLB objective. Thus, the scale changes the planning uncertainty, whereas the true target and the evaluation procedure remain controlled. The terminal RMSE and its degradation relative to the scale-0 reference are reported in Table 6 and Figure 11.

The degradation of both uncertainty-aware methods is consistently smaller than that of the single-point method at every nonzero scale. At the largest tested scale, the terminal RMSE degradation is 61.36% for single-point planning, 47.80% for MC global-CRLB planning, and 40.83% for analytical global-CRLB planning. The analytical method therefore provides a robustness gain of 20.53 percentage points relative to single-point planning. The same trend is observed in the time-averaged RMSE, whose degradation is 19.53% for single-point planning and 13.31% for analytical planning at s=1.00. These results provide a direct robustness check: as the target-position error increases, explicitly accounting for its distribution makes the localization performance degrade more slowly. The result is a sensitivity analysis within the adopted model, rather than a claim of robustness to unmodeled multipath, NLOS bias, or non-Gaussian errors.

**Figure 12:**
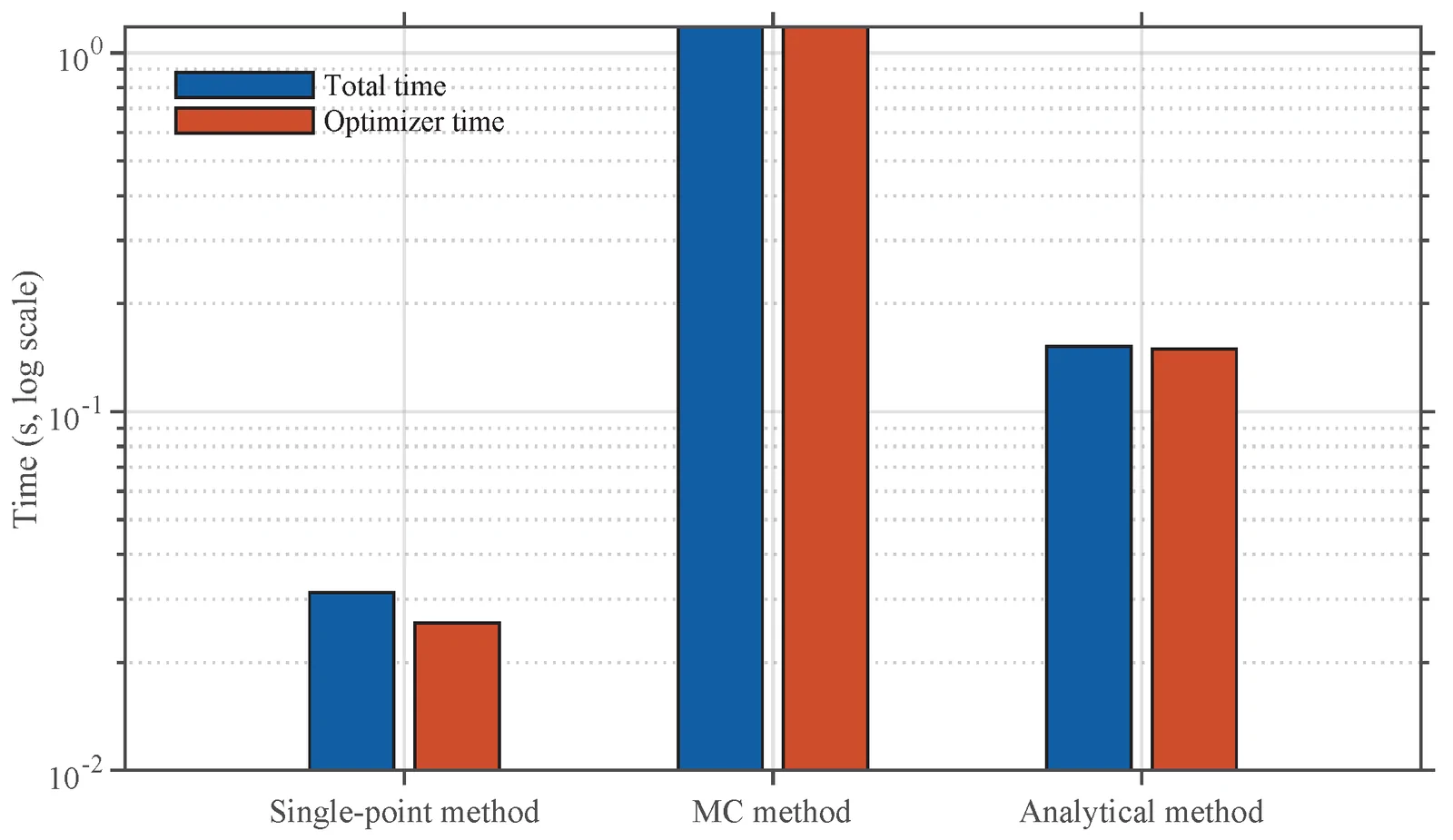
Timing comparison of the three objective evaluations.

Table 7 reports the timing statistics, and Figure 12 visualizes the same timing comparison on a log scale. The MC method has the highest total runtime because it repeatedly samples the target-position uncertainty region. The analytical method reduces the mean total runtime from 1.18225 s to 0.15202 s relative to the MC method, corresponding to an 87.14% reduction and a 7.78-fold speed advantage, while maintaining very similar CRLB and RMSE performance. The single-point method is the fastest, but it does not account for target-position uncertainty in the planning objective.

**Figure 13:**
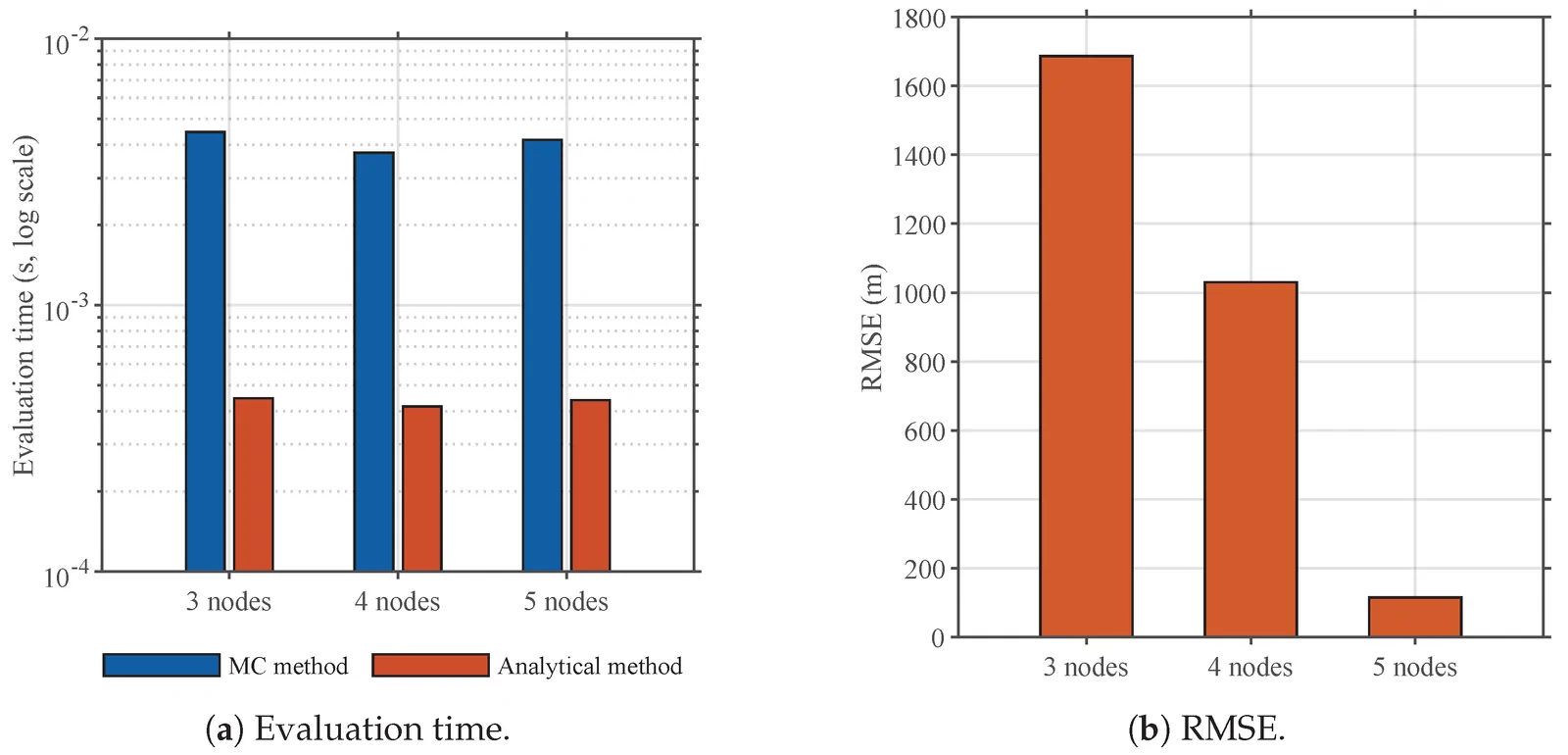
Node-count complexity and RMSE comparison.

To further clarify the computational scaling when additional observation nodes are introduced, Table 8 reports a fixed-geometry 3-, 4-, and 5-node timing comparison at the nominal target-position uncertainty scale, and Figure 13 presents the corresponding time and RMSE comparisons. Figure 13a compares the evaluation time, while Figure 13b compares the RMSE under the same fixed geometry. The comparison includes the CRLB evaluation time, the MC global CRLB evaluation time, and the analytical global CRLB evaluation time. Across the three node-count cases, the analytical global CRLB evaluation remains about one order of magnitude faster than the MC global CRLB reference, while the FIM condition number and localization error are improved by the additional receiver nodes. This comparison characterizes local computational scaling and does not replace the three-UAV main trajectory experiment.

### 5.7. Noise and Node-Count Sensitivity Analysis

**Figure 14:**
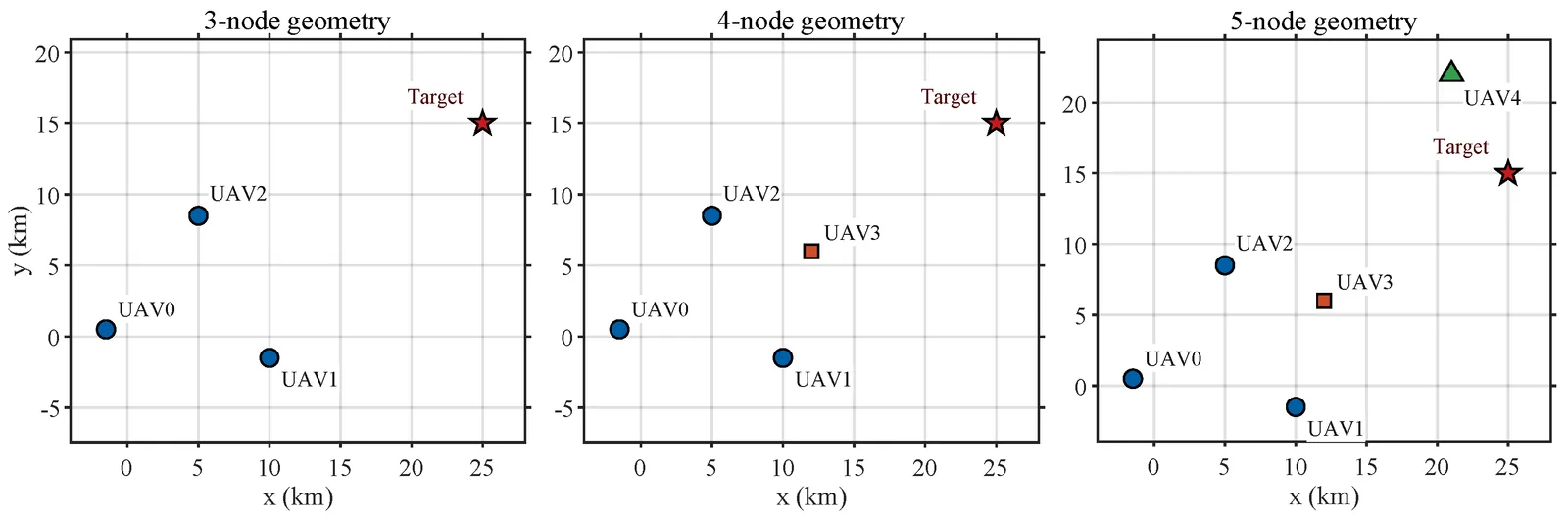
Fixed receiver-node geometries used in the 3-, 4-, and 5-node local sensitivity checks.

**Figure 15:**
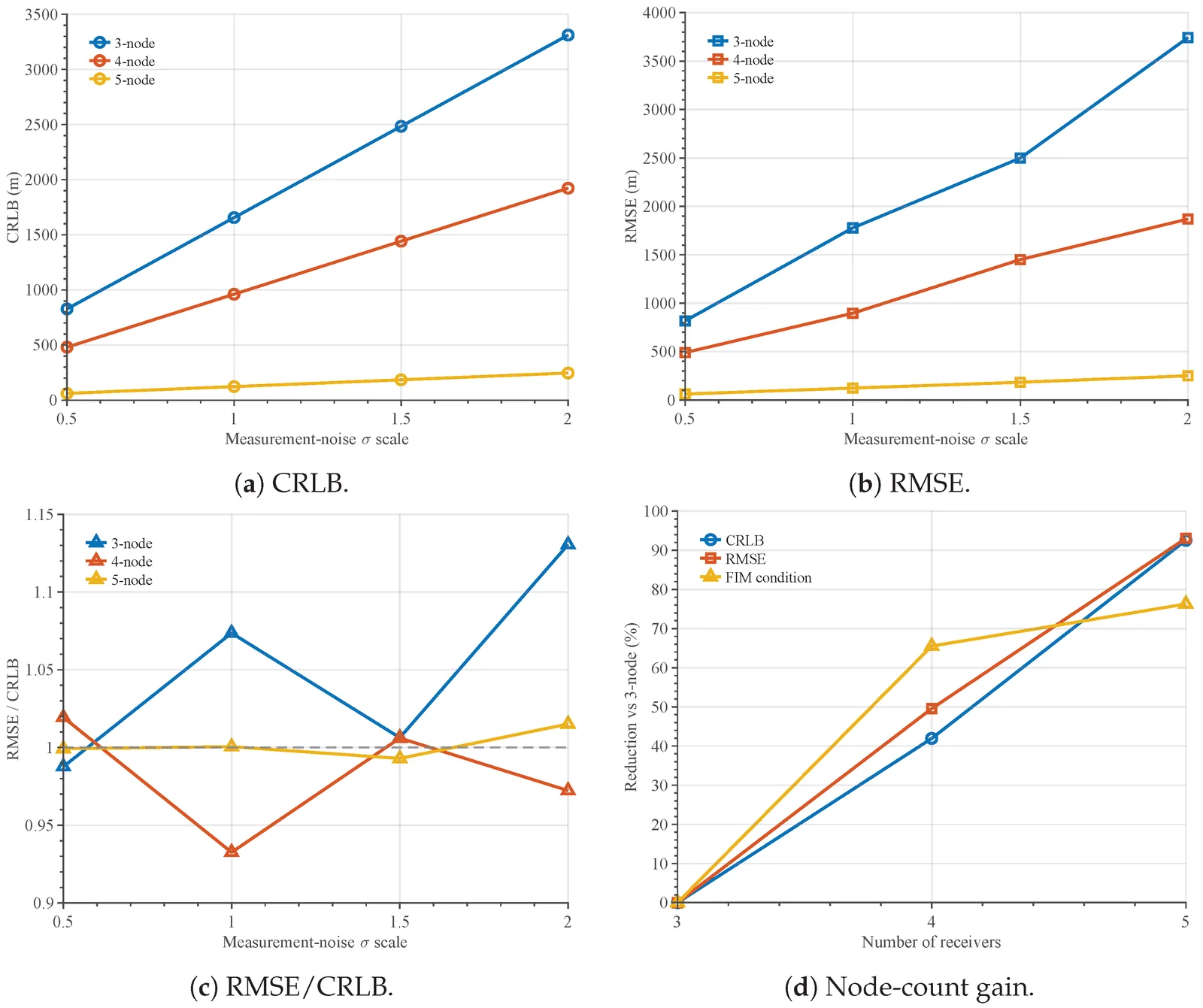
Measurement-noise and node-count sensitivity. In the RMSE/CRLB subpanel, the dotted horizontal line denotes the reference ratio RMSE/CRLB=1.

To evaluate the sensitivity to measurement-noise level and receiver-node number, fixed-geometry experiments are performed under the same CRLB interpretation as the main figures. These experiments characterize measurement-noise and node-count effects without changing the primary three-UAV trajectory-optimization setting. Figure 14 shows the fixed receiver-node geometries used in the 3-, 4-, and 5-node local checks. Figure 15 and Table 9 summarize the measurement-noise sigma sensitivity results, where Figure 15a,b show the CRLB and RMSE changes, and Figure 15c,d show the RMSE/CRLB consistency and node-count gain.

The RMSE-to-CRLB ratios remain close to unity, indicating that the empirical localization errors are consistent with the CRLB scale under the fixed-geometry sensitivity settings; small deviations around unity are mainly attributed to finite Monte Carlo sampling and estimator bias.

**Figure 16:**
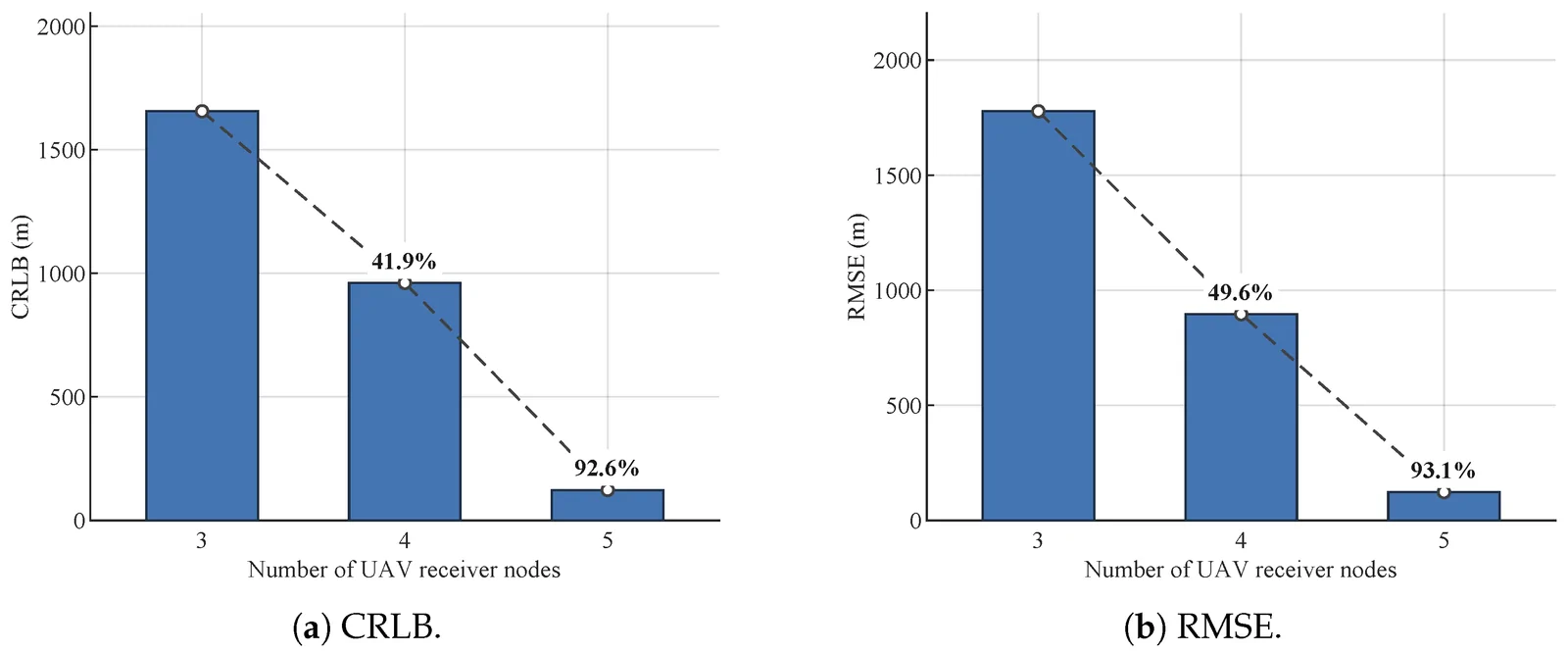
Node-count comparison of CRLB and RMSE at nominal noise.

The sensitivity results show that the CRLB increases with the TDOA noise scale, as expected from the FIM structure. Adding receiver nodes improves the local geometry in this fixed setting and characterizes the node-count effect. At the nominal measurement-noise scale, the separate node-count comparisons in Figure 16, with CRLB in Figure 16a and RMSE in Figure 16b, show that the 4-node case reduces the CRLB and RMSE by 41.94% and 49.57%, respectively, relative to the 3-node case, while the 5-node case reduces them by 92.55% and 93.06%, respectively.

**Figure 17:**
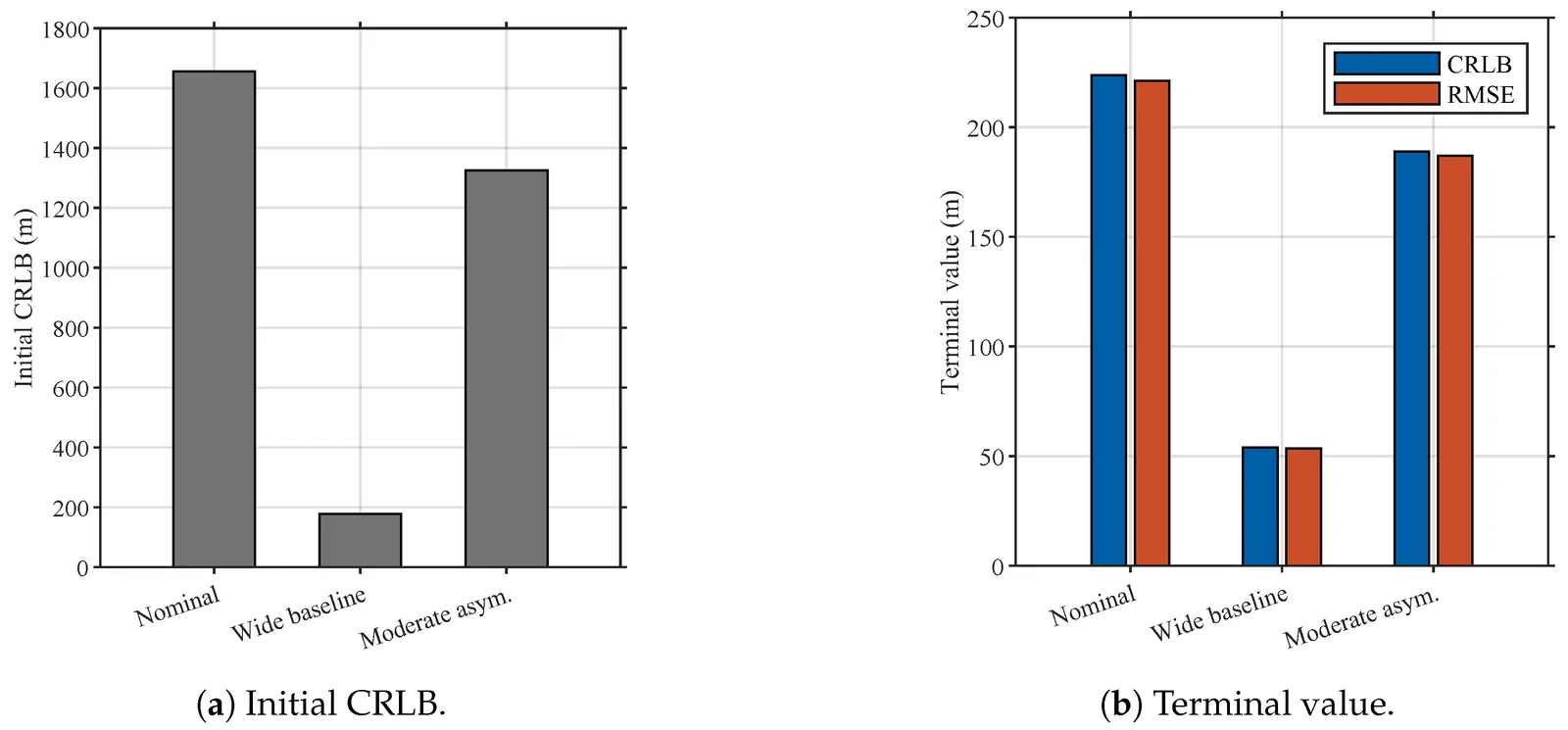
Three-UAV initial-geometry sensitivity.

Finally, to examine the effect of initial observation geometry while keeping the main three-UAV setting unchanged, Table 10 reports a three-UAV initial-geometry sensitivity analysis, and Figure 17 shows the corresponding initial and terminal comparisons. Figure 17a compares the initial CRLB, and Figure 17b compares the terminal CRLB and RMSE. All three cases use the Chan localization algorithm, M=200, 200 target-position samples for the MC global CRLB objective, 30 inner TDOA-noise resamples for RMSE evaluation, and v=50m/s. The results show that the initial FIM condition number and initial CRLB vary substantially with geometry. A favorable wide-baseline geometry gives a much smaller initial CRLB, while a moderately asymmetric geometry provides a nonuniform but non-degenerate perturbation of the nominal observation geometry. In the nominal and moderately asymmetric cases, the analytical terminal CRLB remains close to the MC global CRLB reference while retaining a clear computational advantage. These results characterize the sensitivity to initial observation geometry under the three-UAV setting.

The primary trajectory experiment is the three-UAV experiment in Table 1, Table 2, Table 3, Table 4, Table 5, Table 6 and Table 7 and Figure 4, Figure 5 and Figure 6. The 4- and 5-node results document the fixed-geometry effect of increasing the receiver-node number under the same TDOA noise model and CRLB metric, while Table 10 documents the effect of varying the initial three-UAV observation geometry.

## 6. Conclusions

This paper investigated UAVs’ path optimization for TDOA-based passive localization under target-position uncertainty. A global CRLB objective was formulated to account for the target-position uncertainty distribution, and a second-order analytical approximation was derived to reduce the computational burden of evaluating this objective inside a PSO-based planner. The three-UAV main experiment uses a practical UAV speed of 50 m/s, Chan localization, CRLB as the unified displayed CRLB metric, and RMSE as the localization-error metric.

The numerical results show that the MC method and analytical method both improve the CRLB and RMSE relative to the single-point method in the main three-UAV scenario. The analytical method remains close to the MC method, with a mean absolute relative difference of 1.62% in CRLB and 1.67% in RMSE, while reducing the mean total runtime from 1.18225 s to 0.15202 s relative to the MC method. The target-position-error sweep further shows that, at the largest tested uncertainty scale, the terminal RMSE degradation is 40.83% for analytical planning compared with 61.36% for single-point planning. Fixed-geometry sensitivity analyses further show the expected sensitivity to measurement noise, the benefit of additional receiver nodes, and the dependence on initial three-UAV observation geometry.

Future work will extend the framework to full 3D trajectory optimization, richer UAV dynamics, and broader external planner comparisons.

## Figures and Tables

**Table 1:** Main simulation parameters for the three-UAV experiment.

Parameter	Value
Number of UAVs	3
Localization algorithm	Chan
UAV speed	50 m/s
Planning interval	10 s
Movement radius per step	500 m
Outer Monte Carlo runs for mean/CI	200
Global CRLB MC target-position samples	200
RMSE inner TDOA-noise resamples	30
PSO particle number/maximum iterations	40/15
Displayed CRLB metric	CRLB evaluated at the true target position
Localization metric	RMSE

**Table 2:** CRLB and RMSE numerical comparisons between three path optimization algorithms. The reported confidence intervals are 95% confidence intervals at the terminal time step.

Method	Mean CRLB	Final CRLB	CI95	Mean RMSE	Final RMSE	CI95
Single-point method	662.05	241.62	1.43	667.65	239.20	14.52
MC method	589.03	224.80	1.14	592.07	222.49	13.59
Analytical method	601.63	223.72	1.27	605.34	221.24	12.97

**Table 3:** Solver parameters for the three-UAV optimizer comparison.

Parameter	PSO	GA	ACO
Candidate number	40 particles	40 individuals	40 ants
Iteration setting	15 iterations	15 generations	15 iterations
Shared method setting	Traditional method/single-point method/MC method/analytical method	Same as PSO	Same as PSO
Special parameters	Shared random seed	Continuous bounded-heading search	Evaporation 0.35/elite fraction 0.25
Optimization strategy	Bounded heading optimization with common trajectory constraints	Bounded heading optimization with common trajectory constraints	Bounded heading optimization with common trajectory constraints

**Table 4:** Relative difference between the analytical method and the MC method.

Metric	Mean Abs. Diff. (%)	Max Abs. Diff. (%)	Final Diff. (%)
CRLB	1.62	3.43	−0.48
Global CRLB objective	3.66	12.42	0.37
RMSE	1.67	3.55	−0.56

**Table 5:** Target-position uncertainty-scale analysis for the analytical global CRLB approximation. The uncertainty scale refers to the target-position uncertainty, not to TDOA measurement-noise standard deviation.

Uncertainty Scale	Mean Abs. Diff. (%)	Max Abs. Diff. (%)	Mean Time Ratio	Interpretation
0.25	0.0867	0.0936	14.04×	Primary accuracy range
0.50	1.8186	3.7193	14.84×	Primary accuracy range
0.75	3.3792	4.8135	10.47×	Primary accuracy range
1.00	6.2287	16.0896	9.49×	Broad-uncertainty stress case

**Table 6:** Robustness to increasing target-position error. Each method column reports terminal RMSE (m)/degradation (%) relative to its corresponding value at s=0; the robustness gain is the single-point degradation minus the analytical degradation.

Scale *s*	Single-Point RMSE/Degradation	MC Global CRLB RMSE/Degradation	Analytical Global CRLB RMSE/Degradation	Analytical Robustness Gain (Percentage Points)
0.00	237.510/0.00	237.510/0.00	237.510/0.00	0.00
0.25	239.662/0.91	239.174/0.70	239.130/0.68	0.22
0.50	253.214/6.61	245.187/3.23	245.244/3.26	3.36
0.75	281.314/18.44	262.120/10.36	261.637/10.16	8.28
1.00	383.250/61.36	351.036/47.80	334.489/40.83	20.53

**Table 7:** Timing comparison of the three PSO-based objectives in the three-UAV main experiment.

Method	CRLB Time (s)	Opt. Time (s)	Total Mean (s)	Total Max (s)
Single-point method	0.00478	0.02580	0.03131	2.26727
MC method	0.00184	1.17992	1.18225	3.09303
Analytical method	0.00211	0.14938	0.15202	0.35784

**Table 8:** Fixed-geometry multi-node computational-complexity comparison at the nominal target-position uncertainty scale.

Nodes	CRLB Time	MC Time	Analytical Time	Time Ratio	FIM Cond.	RMSE
3	0.001147	0.004469	0.000447	9.99	345.83	1686.79
4	0.000149	0.003729	0.000416	8.97	119.19	1029.87
5	0.000044	0.004178	0.000440	9.49	82.04	115.32

**Table 9:** Fixed-geometry CRLB and RMSE sensitivity to measurement-noise scale for 3-, 4-, and 5-node configurations.

Noise Scale	Factor	3-Node CRLB	4-Node CRLB	5-Node CRLB	3-Node RMSE	4-Node RMSE	5-Node RMSE
0.5	100	827.79	480.62	61.66	817.57	489.93	61.61
1.0	200	1655.58	961.23	123.32	1777.46	896.42	123.39
1.5	300	2483.37	1441.85	184.98	2499.05	1450.57	183.68
2.0	400	3311.16	1922.46	246.64	3743.33	1869.20	250.36

**Table 10:** Three-UAV initial-geometry sensitivity analysis. The table reports the analytical global CRLB terminal result and its relative difference from the MC global CRLB terminal CRLB.

Initial Geometry	Initial FIM Cond.	Initial CRLB	Analytical Final CRLB	Analytical Final RMSE	Analytical vs. MC Diff.	Analytical Speedup
		(m)	(m)	(m)	(%)	vs. MC
Nominal current	345.83	1655.58	223.72	221.24	−0.48	8.19×
Favorable wide-baseline	53.19	177.20	53.92	53.52	8.74	8.27×
Moderately asymmetric	271.08	1325.73	188.98	187.03	−0.71	7.70×

## Data Availability

Due to privacy constraints, we are unable to share the data utilized in this study for submission purposes. We assure you that the research methodology and results outlined in this paper comply with ethical guidelines and have been executed in line with recognized research protocols.
